# Nearly Exact Discrepancy Principle for Low-Count Poisson Image Restoration

**DOI:** 10.3390/jimaging8010001

**Published:** 2021-12-23

**Authors:** Francesca Bevilacqua, Alessandro Lanza, Monica Pragliola, Fiorella Sgallari

**Affiliations:** Department of Mathematics, University of Bologna, Piazza di Porta San Donato 5, 40126 Bologna, Italy; francesca.bevilacqu8@unibo.it (F.B.); alessandro.lanza2@unibo.it (A.L.); fiorella.sgallari@unibo.it (F.S.)

**Keywords:** image restoration, Poisson noise, discrepancy principle, alternating direction method of multipliers

## Abstract

The effectiveness of variational methods for restoring images corrupted by Poisson noise strongly depends on the suitable selection of the regularization parameter balancing the effect of the regulation term(s) and the generalized Kullback–Liebler divergence data term. One of the approaches still commonly used today for choosing the parameter is the discrepancy principle proposed by Zanella et al. in a seminal work. It relies on imposing a value of the data term approximately equal to its expected value and works well for mid- and high-count Poisson noise corruptions. However, the series truncation approximation used in the theoretical derivation of the expected value leads to poor performance for low-count Poisson noise. In this paper, we highlight the theoretical limits of the approach and then propose a nearly exact version of it based on Monte Carlo simulation and weighted least-square fitting. Several numerical experiments are presented, proving beyond doubt that in the low-count Poisson regime, the proposed modified, nearly exact discrepancy principle performs far better than the original, approximated one by Zanella et al., whereas it works similarly or slightly better in the mid- and high-count regimes.

## 1. Introduction

The image restoration problem under Poisson noise corruption is a task that has been extensively addressed in the literature as it arises in many real-world applications, where the acquired image is obtained by counting the particles, e.g., photons, hitting the image domain [1]. The typical image formation model under blur and Poisson noise corruption takes the form

(1) $$y\;\;=\;\;\mathrm{Poiss}\left(\lambda \right)\;,\;\;\lambda =H\bar{x}+b\;,$$

where $H\in R^{m\times n}$ models the blur operator, which we assume to be known; $\;y\in N^{m}$ and $\bar{x}\in R^{n}$ are the observed $m_{1}\times m_{2}$ and unknown $n_{1}\times n_{2}$ images in column-major vectorized form (with $m=m_{1}m_{2}$ and $n=n_{1}n_{2}$), respectively; $b\in R^{m}$ is a non-negative background emission, and where $\;\mathrm{Poiss}\left(\lambda \right):={\left(\mathrm{Poiss}\left(\lambda_{1}\right),\ldots ,\mathrm{Poiss}\left(\lambda_{m}\right)\right)}^{T}$, with $\mathrm{Poiss}(\lambda_{i})$ indicating the realization of a Poisson-distributed random variable of parameter (mean) $\lambda_{i}$.

When tackling the recovery of $\bar{x}$ starting from y, one has also to consider the intrinsic constraint

(2) $$\bar{x}\;\;\in \;\;\Omega \;\;:=\;\;\left\{\bar{x}\;\in \;R^{n}\;:\;\;\bar{x}\;\geq \;0\right\}\;,$$

which accounts for the pixel values being non-negative.

In a probabilistic perspective [2], problem (1) and (2) can be addressed by modeling the unknown x as a random variable. In general, the information on the degradation process is encoded in the so-called *likelihood* probability density function (pdf) p(y∣x), while the prior beliefs on the unknown x are expressed by the *prior* pdf p(x). In the Bayesian framework, one aims to recover the *posterior* pdf, which is related to the likelihood and the prior term via the Bayes formula:

p(x∣y)∝P(y∣x)p(x).

with *P* denoting the probability mass function (pmf) that replaces the continuous pdf *p* to account for the discrete nature of the data y.

According to the Maximum A Posteriori (MAP) estimation approach, the mode of p(x∣y) can be selected as a single-point representative of the posterior distribution, so that the original problem (1) and (2) turns into:

(3) $$\begin{array}{cc} x^{*}\;\in \; & \underset{x}{arg max}\left\{\;p(y|x)\;p(x)\;\right\}\\ \;=\; & \underset{x}{arg min}\left\{\;-\ln p(y|x)\;-\;\ln p(x)\;\right\}\;. \end{array}$$

In light of the constraint expressed in (2), the general form of the prior pdf reads

$$p\left(x\right)\propto p_{c}\left(x\right)p_{0}\left(x\right)\;,$$

with

$$p_{c}\left(x\right)=\left\{\begin{array}{cc} 1 & \mathrm{if}\;x\in \Omega \\ 0 & \mathrm{otherwise} \end{array}\right.\;,$$

and $p_{0}\left(x\right)$ encoding other information possibly available on x. A typical choice for $p_{0}\left(x\right)$ is given by the Total Variation (TV) Gibbs prior—see [3]—which reads

$$p_{0}\left(x\right)=\frac{1}{Z}\exp\left(-\alpha \sum_{i=1}^{n}{∥{\left(Dx\right)}_{i}∥}_{2}\right)\;,$$

where Z>0 is a normalization constant, α>0 is the prior parameter and $D:={\left(D_{h}^{T},D_{v}^{T}\right)}^{T}\in R^{2n\times n}$ denotes the discrete gradient operator with $D_{h},D_{v}\in R^{n\times n}$, two linear operators representing the finite difference discretizations of the first-order partial derivatives of the image x in the horizontal and vertical direction. The negative logarithm of the prior pdf p(x) thus reads

(4) $$-\ln p\left(x\right)=\alpha \sum_{i=1}^{n}{∥{\left(Dx\right)}_{i}∥}_{2}+\;\iota_{\Omega }\left(x\right)\;+\;\ln Z\;,$$

with $\iota_{\Omega }\left(\cdot \right)$ denoting the indicator function of set Ω, which is equal to 0 if x∈Ω, or +∞ otherwise.

Concerning the likelihood pdf, first, we notice that the forward model (1) can be usefully rewritten in component-wise (pixel-wise) form as follows:

$$y_{i}\;\;=\;\;\mathrm{Poiss}\left(\lambda_{i}\right)\;,\;\lambda_{i}=H_{i}x+b_{i}\;,\;\;i=1,\ldots ,m\;,$$

with $y_{i}\in N$, $\;\lambda_{i},b_{i}\in R_{+}\;$, and where $H_{i}\in R^{1\times n}$ denotes the *i*-th row of matrix H. Upon the assumption of independence of the Poisson noise realizations at different pixels, we have:

(5) $$\begin{array}{c} \begin{array}{cc} -\ln P\left(y|x\right)=-\ln P\left(y|\lambda \right)\;=\; & -\ln\prod_{i=1}^{m}P\left(y_{i}|\lambda_{i}\right)\\ \;=\; & -\sum_{i=1}^{m}\ln P\left(y_{i}|\lambda_{i}\right)\;,\;\lambda =Hx+b\;, \end{array} \end{array}$$

where $P\left(y_{i}|\lambda_{i}\right)$, which denotes the probability for $y_{i}$ to be the realization of a Poisson-distributed random variable with mean $\lambda_{i}$, reads

$$P\left(y_{i}|\lambda_{i}\right)\;\;=\;\frac{\lambda_{i}^{y_{i}}\;e^{-\lambda_{i}}}{y_{i}!}\;,\;\;y_{i}\in N,\;\;\lambda_{i}\in R_{+}\;,$$

with $R_{+}$ denoting the set of non-negative real numbers. Hence, the associated negative log-pmf reads

(6) $$-\ln P\left(y_{i}|\lambda_{i}\right)\;\;=\;\;\lambda_{i}\;-\;y_{i}\;\ln\lambda_{i}\;+\;\ln\left(y_{i}!\right)\;.$$

By plugging (6) into (5), the negative log-likelihood takes the following form:

(7) $$-\ln P\left(y|x\right)\;=\;\sum_{i=1}^{m}\left(\;\lambda_{i}\;-\;y_{i}\ln\lambda_{i}\;+\;\ln\left(y_{i}!\right)\;\right)\;.$$

Finally, plugging (4) and (7) into (3), dropping out the constant term lnZ in (4), readjusting the constant terms in (7) (by adding $\;y_{i}\ln y_{i}-y_{i}-\ln\left(y_{i}!\right)\;$ to each term in the sum), and then dividing the cost function by the positive scalar α, we obtain the so-called TV-KL variational model:

(8) $$\hat{x}\left(\mu \right)\;=\;\underset{x\;\in \;\Omega }{arg min}\;\left\{\;J\left(x;\mu \right)\;:=\;\mathrm{TV}\left(x\right)\;+\;\mu \;\mathrm{KL}\left(\lambda ;y\right)\;\right\},\;\lambda =Hx+b\;,$$

where μ=1/α, the TV semi-norm term [4], is defined by

$$\mathrm{TV}\left(x\right)\;\;=\;\;\sum_{i=1}^{n}{∥{\left(Du\right)}_{i}∥}_{2}\;,$$

and the term $\;\mathrm{KL}\left(\lambda ;y\right)\;$ indicates the generalized Kullback–Leibler (KL) divergence between λ=Hx+b and the observation y, which reads

(9) $$\mathrm{KL}\left(\lambda ;y\right)\;\;=\;\;\sum_{i=1}^{m}F\left(\lambda_{i};y_{i}\right)\;,\;\;\;\mathrm{with}\;\;\;F\left(\lambda ;y\right)\;\;:=\;\;\lambda \;-\;y\ln\lambda \;+\;y\ln y\;-\;y\;.$$

Note that the adoption of the MAP strategy within a probabilistic framework yields a minimization problem, which is typically addressed in the context of variational methods for image restoration. The TV term and the KL divergence play the role of the *regularization* and *data fidelity term*, respectively. Moreover, the parameter μ, which has been defined starting from the prior parameter α, is the so-called *regularization parameter* balancing the action of regularization and data fidelity terms.

The selection of a suitable value for the regularization parameter μ is of crucial importance for obtaining high-quality results. This relation is highlighted by the explicit dependence in (8) of the solution $\hat{x}$ on the parameter μ. Very often, μ is chosen empirically by brute-force optimization with respect to some visual quality metrics. However, for Poisson data, a large amount of literature has been devoted to the analysis of the Discrepancy Principle (DP), which can be formulated in general terms as follows [5]:

(10) $$\mathrm{Select}\;\;\;\mu \;=\;\mu^{*}\in R_{+}\;\;\mathrm{such}\;\;\mathrm{that}\;\;\;D\left(\mu^{*};y\right)\;=\;\Delta \;,$$

where the last equality and the scalar $\Delta \in R_{+}$ in (10) are commonly referred to as the *discrepancy equation* and the *discrepancy value*, respectively, while the *discrepancy function*$\;D\left(\;\cdot \;;y\right):R_{+}\to R_{+}$ is defined by

(11) $$D\left(\mu ;y\right)\;\;:=\;\;\mathrm{KL}\left(\;\hat{\lambda }\left(\mu \right);y\right)\;\;=\;\;\sum_{i=1}^{m}\left(D_{i}\left(\mu ;y_{i}\right)\;:=\;F\left({\hat{\lambda }}_{i}\left(\mu \right);y_{i}\right)\right)\;,$$

with function *F* defined in (9) and

(12) $$\hat{\lambda }\left(\mu \right)\;=\;H\;\hat{x}\left(\mu \right)+b\;.$$

The DP in (10)–(12) formalizes a quite simple idea: choose the value $\mu^{*}$ of the regularization parameter μ in the TV-KL model (8) such that the value of the KL data fidelity term associated with the solution $\hat{x}\left(\mu^{*}\right)$ is equal to a prescribed discrepancy value Δ. However, applying the DP in an effective manner in practice is not straightforward as several issues concerning the computational efficiency and, more importantly, the quality of the output solutions arise. We examine both of them more closely.

(I1) *Computational efficiency.* The solution function $\;\hat{x}\left(\mu \right)$ of model (8) does not admit a closed-form expression and iterative solvers must be used to compute the restored image $\hat{x}$ associated with any μ. Hence, selecting $\mu^{*}$ by solving the scalar discrepancy equation defined in (10)–(12) as an efficient preliminary step and then computing the sought restored image $\hat{x}\left(\mu^{*}\right)$ by iteratively solving model (8) only once is not feasible.
(I2) *Quality of solution(s).* Even if an efficient algorithm is used for the computation, the obtained restored image $\;\hat{x}\left(\mu^{*}\right)\;$ may be of such low quality that it is of no practical use if the discrepancy value Δ in (10) is not suitably chosen.

Issue (I1) concerning computational efficiency has been successfully addressed in [6], where the authors propose to automatically update μ along the iterations of the minimization algorithm used for solving the TV-KL model so as to satisfy (at convergence) a specific version of the general DP defined in (10)–(12).

Concerning (I2), we highlight that, in the theoretical hypothesis that the target image $\bar{x}$ is known, so that $\bar{\lambda }=H\bar{x}+b$ is also known, one would select $\mu^{*}$ such that the value of the KL fidelity term associated with the solution $\hat{x}\left(\mu^{*}\right)$ is equal to the value of the KL fidelity term associated with $\bar{x}$. This clearly does not guarantee that the obtained solution $\hat{x}\left(\mu^{*}\right)$ coincides with the target image $\bar{x}$. However, by constraining $\hat{x}\left(\mu \right)$ to belong to the unique level set of the (convex) KL fidelity term containing $\bar{x}$, this abstract strategy, which we refer to as the Theoretical DP (TDP), represents an oracle for the general DP in (10)–(12). The TDP is thus formulated as follows:

(13) $$\begin{array}{c} \begin{array}{cc} \; & \mathrm{Select}\;\;\;\mu \;=\;\mu^{*}\in R_{+}\;\;\mathrm{such}\;\;\mathrm{that}\;\;\;D\left(\mu^{*};y\right)\;=\;\Delta^{\left(T\right)}\;,\\ \; & \mathrm{with}\;\;\Delta^{\left(T\right)}\;:=\sum_{i=1}^{m}\;\left(\delta^{\left(T\right)}\left({\bar{\lambda }}_{i}\right)\;:=\;F\left({\bar{\lambda }}_{i}\;;\;y_{i}\right)\right),\;\;\;\bar{\lambda }\;=\;H\bar{x}+b\;, \end{array} \end{array}$$

with function *F* defined in (9). Clearly, the value $\Delta^{\left(T\right)}$ cannot be computed in practice as the original image $\bar{x}$ is not available. As in the case of the Morozov discrepancy principle for Gaussian noise, one could replace the scalar $\Delta^{\left(T\right)}$ with the expected value of the KL fidelity term in (9) regarded as a function of the *m*-variate random variable Y. We will refer to this version of the DP as Exact (or Expected value) DP (EDP). In the following formula

(14) $$\begin{array}{c} \begin{array}{cc} \; & \mathrm{Select}\;\;\;\mu \;=\;\mu^{*}\in R_{+}\;\;\mathrm{such}\;\;\mathrm{that}\;\;\;D\left(\mu^{*};y\right)\;=\;\Delta^{\left(E\right)}\left(\mu^{*}\right)\;,\\ \; & \mathrm{with}\;\;\Delta^{\left(E\right)}\left(\mu \right)\;:=\sum_{i=1}^{m}\;\left(\delta^{\left(E\right)}\left({\hat{\lambda }}_{i}\left(\mu \right)\right)\;:=\;E_{Y_{i}}\left[F\left({\hat{\lambda }}_{i}\left(\mu \right);Y_{i}\right)\right]\right),\;\;\;\hat{\lambda }\left(\mu \right)\;=\;H\hat{x}\left(\mu \right)+b\;, \end{array} \end{array}$$

where $E_{Y_{i}}\left[F\left({\hat{\lambda }}_{i}\left(\mu \right);Y_{i}\right)\right]$ denotes the expected value of $F\left({\hat{\lambda }}_{i}\left(\mu \right);Y_{i}\right)$ regarded as a function of the Poisson-distributed random variable $Y_{i}$. Nonetheless, unlike the Gaussian noise case, the discrepancy value is not a constant but is a function $\Delta^{\left(E\right)}\left(\mu \right)$ of the regularization parameter μ, and deriving its analytic expression is a very challenging task. A popular and widespread strategy, originally proposed in [5] for denoising purposes and extended in [7] to the image restoration task, replaces the exact expected value function $\Delta^{\left(E\right)}\left(\mu \right)$ with a constant approximation coming from truncating its Taylor series expansion. We will refer to this version of the DP as Approximate DP (ADP). It reads:   

(15) $$\begin{array}{c} \begin{array}{cc} \; & \mathrm{Select}\;\;\;\mu \;=\;\mu^{*}\in R_{+}\;\;\mathrm{such}\;\;\mathrm{that}\;\;\;D\left(\mu^{*};y\right)\;=\;\Delta^{\left(A\right)}\;,\\ \; & \mathrm{with}\;\;\Delta^{\left(A\right)}\;:=\sum_{i=1}^{m}\left(\delta^{\left(A\right)}\;:=\;\frac{1}{2}\right)\;=\;\frac{m}{2}. \end{array} \end{array}$$

**Figure 1:**
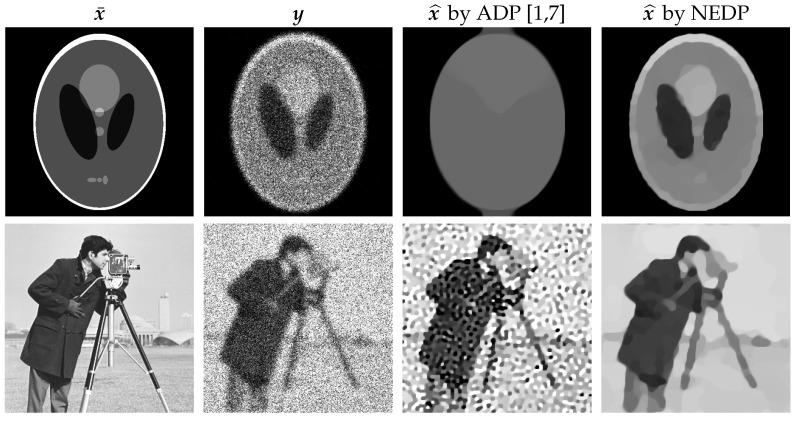
Original images (**first** column), observed images corrupted by blur and Poisson noise (**second** column), restored images obtained by the TV-KL model (8) and (9) with μ selected according to the approximate DP [5,7] (**third** column), and the proposed nearly exact DP (**last** column).

Despite its extensive use due to the good performance achieved in the mid- and high-count regimes, the (15) is known to return poor-quality results in the low-count Poisson regime [8], i.e., when the number of photons hitting the image domain is small. In fact, in [7], where the ADP was first extended to the image deblurring task, the authors state (in Remark 3) that the choice of the constant value $\delta^{\left(A\right)}=1/2$ in (15) may not be “optimal” and suggest replacing it with 1/2+ϵ, where ϵ is a small positive or negative real number. As a preliminary, qualitative proof of the possible poor performance of (15) in the low-count regime, in the first column of Figure 1, we show the two test images phantom and cameraman, which have been corrupted by blur and heavy Poisson noise (second column). The TV-KL image restoration model in (8), with regularization parameter μ selected according to the (15), has been performed. The output restorations are displayed in the third column of Figure 1. One can see that the rough approximation $\delta^{\left(A\right)}=1/2$ used in the (15) can either return oversmoothed results, as in the case of phantom, or undersmoothed restorations, as for cameraman.

Since its proposal in [5], the ADP has been (and still is) widely used for variational image restoration (see, e.g., [9,10]) and it can be regarded as the standard extension of the Morozov DP for Gaussian noise to the Poisson noise case. Then, some literature exists working on the ADP, e.g., by proposing, analyzing, and testing its usage in KL-constrained variational models [11] or by analyzing it theoretically [12]. However, to the best of the authors’ knowledge, the only attempt to improve the ADP by giving a face to the ϵ adjustment to the approximate, constant discrepancy value $\;\delta^{\left(A\right)}=1/2\;$ is the one in [8]. The authors in [8] correctly state that ϵ must not be a constant, but a function ϵ(λ) of the photon count level. However, they propose to take ϵ(λ) as the sum of the second to tenth terms of the same Taylor expansion used in [5]. As we will highlight later in the paper, such expansion converges only for λ approaching +∞; hence, the choice in [8] cannot aspire to improve the performance of ADP in low-count regimes.

### Contribution

The goal of this paper is to provide novel insights about the EDP and the ADP in order to design a novel discrepancy principle capable of outperforming the classical ADP proposed in [5]. In more detail, we will provide a qualitative study proving that the recovery of a closed-form expression for function $\Delta^{\left(E\right)}\left(\mu \right)$ in (14) through its Taylor series expansion used in [5,8] is not only difficult to achieve but also theoretically unfeasible for low-count Poisson regimes. Moreover, we will explore in detail the properties of the ADP motivating the dichotomic behavior (i.e., oversmoothing/undersmoothing) arising upon its adoption in the low-count regime. Finally, based on a simple Monte Carlo simulation followed by weighted least-square fitting, we will derive a novel version of the general DP in (10)–(12) based on a nearly exact (NE) approximation of function $\Delta^{\left(E\right)}\left(\mu \right)$ in (14), concisely referred to as NEDP. Our approach will successfully address issues (I1)–(I2). In particular, it will be demonstrated experimentally that NEDP can return high-quality results both for low-count and mid/high-count acquisitions. The good performance of NEDP is anticipated in the last column of Figure 1, where we show the output restorations achieved by using the TV-KL model in (8) and (9) coupled with the novel μ-selection strategy.

## 2. Limits of the Approximate DP

The discrepancy principle proposed by Zanella et al. in [5] for Poisson image denoising and then extended to image restoration by Bertero et al. in [7] relies on Lemma 1 in [5], whose proof has been completed in [5] (corrigendum), which we report below for completeness.

**Lemma** **1.** *Let $Y_{\lambda }$ be a Poisson random variable with expected value $\lambda \in R_{++}$ and consider the function of $\;Y_{\lambda }$ defined by*

(16) $$F\left(Y_{\lambda }\right)\;=\;\lambda -Y_{\lambda }\ln\lambda +Y_{\lambda }\ln Y_{\lambda }-Y_{\lambda }\;=\;Y_{\lambda }\ln\left(1+\frac{Y_{\lambda }-\lambda }{\lambda }\right)+\lambda -Y_{\lambda }\;.$$

*Then, the following estimate of the expected value of $F(Y_{\lambda })$ holds true for large λ:*

(17) $$\delta^{\left(E\right)}\left(\lambda \right)\;\;=\;\;E\left\{F\left(Y_{\lambda }\right)\right\}\;\;=\;\;\delta^{\left(A\right)}+O\left(\frac{1}{\lambda }\right)\;,\;\;\;\delta^{\left(A\right)}\;=\;\frac{1}{2}\;.$$

Based on the estimate above, and implicitly assuming a sufficiently large λ (i.e., a sufficiently high-count Poisson regime) such that the O(1/λ) term can be neglected, the exact DP outlined in (14) is replaced in [5,7] by the approximation given in (15) and recalled below:

$$\Delta \;\;=\;\;\Delta^{\left(A\right)}\;\;=\;\;\sum_{i=1}^{m}\delta^{\left(A\right)}\;\;=\;\;\frac{m}{2}\;.$$

However, the ADP performs poorly for low-count Poisson images. Our goal here is to highlight that the reason for this lies precisely in the constant approximation $\;\delta^{\left(E\right)}\left(\lambda \right)\approx \delta^{\left(A\right)}\;$ used in (15) and then propose a nearly exact DP based on a much less approximate estimate $\;\delta^{\left(NE\right)}\left(\lambda \right)\;$ of the expected value function $\;\delta^{\left(E\right)}\left(\lambda \right)\;$.

**Figure 2:**
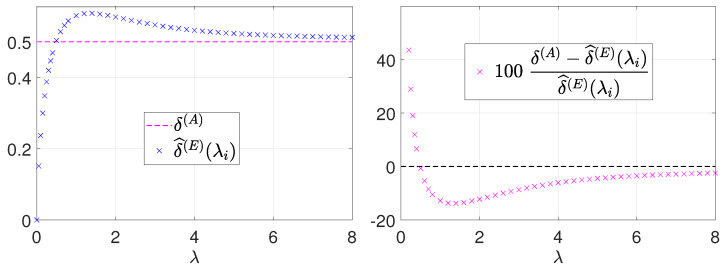
Comparison between the approximation $\;\delta^{\left(A\right)}=1/2\;$ of $\;\delta^{\left(E\right)}\left(\lambda \right)=E\left\{F\left(Y_{\lambda }\right)\right\}\;$ used in the (15) proposed in [5,7] and the Monte Carlo estimates $\;{\hat{\delta }}^{\;(E)}\left(\lambda_{i}\right)\;$ for some $\lambda_{i}\in \left[0,8\right]$.

For this purpose, first, we carry out a preliminary Monte Carlo simulation aimed at highlighting the error associated with the approximation in (15). In particular, we consider a discrete set of λ values $\lambda_{i}\in \left[0,8\right]$ and, for each $\lambda_{i}$, we generate pseudo-randomly a large number ($10^{6})$ of realizations of the Poisson random variable $Y_{\lambda_{i}}$. Then, we compute the associated values of the function $F(Y_{\lambda_{i}})$ defined in (16) and, finally, for each $\lambda_{i}$, we obtain an estimate $\;{\hat{\delta }}^{\left(E\right)}\left(\lambda_{i}\right)\;$ of $\;\delta^{\left(E\right)}\left(\lambda_{i}\right)\;$ by calculating the sample mean of these function values. The results of this simulation are shown in Figure 2. In particular, in the left figure, we report the computed estimates $\;{\hat{\delta }}^{\left(E\right)}\left(\lambda_{i}\right)\;$, whereas, in the right figure, we report the percentage errors (with respect to the estimates) associated with using the constant value $\;\delta^{\left(A\right)}=1/2$ as in the (15). The percentage error approaches +∞ for λ tending to zero and is in the order of −10% for λ∈[1,4]; then, as expected, it decreases (quite slowly) to zero for λ tending to +∞. The error is thus quite large for small λ and this can explain the poor performance of the (15) in the low-count Poisson regime.

In order to obtain a more accurate approximation or even an exact analytical expression for the expected value function $\;\delta^{\left(E\right)}\left(\lambda \right)\;$, we now retrace in detail the proof of Lemma 1 given in [5], and completed in [5] (corrigendum), and check if the rough truncation carried out in [5] can be avoided.

After noting that function ln(1+φ) is $C^{\infty }$ on its domain (−1,+∞) and considering its Taylor expansion around 0, the Taylor theorem with the remainder in integral form allows one to write:

(18) $$\begin{array}{ccc} \ln\left(1+\varphi \right) & \;\;\;\;=\;\;\;\; & \sum_{i=1}^{N}\frac{{\left(-1\right)}^{i+1}}{i}\;\varphi^{i}+r_{N}\left(\varphi \right)\;=\;\varphi -\frac{1}{2}\varphi^{2}+\frac{1}{3}\varphi^{3}-\cdots +\frac{{\left(-1\right)}^{N+1}}{N}\varphi^{N}+r_{N}\left(\varphi \right),\\ r_{N}\left(\varphi \right) & \;\;\;\;=\;\;\;\; & {\left(-1\right)}^{N}\int_{0}^{\varphi }\frac{{\left(\varphi -t\right)}^{N}}{{\left(1+t\right)}^{N+1}}\;dt\;,\;\;\forall \;\varphi \in \left(-1,+\infty \right)\;. \end{array}$$

Replacing the expansion above with $\varphi =(Y_{\lambda }-\lambda )/\lambda$ in the expression of function *F* defined in (16), we obtain

(19) $$\begin{array}{ccc} F\left(Y_{\lambda }\right) & = & Y_{\lambda }\left(\sum_{i=1}^{N}\frac{{\left(-1\right)}^{i+1}}{i}\;{\left(\frac{Y_{\lambda }-\lambda }{\lambda }\right)}^{i}+r_{N}\left(\varphi \right)\right)+\lambda -Y_{\lambda }\\ & = & Y_{\lambda }r_{N}\left(\varphi \right)+\left(Y_{\lambda }-\lambda +\lambda \right)\left(\sum_{i=1}^{N}\frac{{\left(-1\right)}^{i+1}}{i}\;{\left(\frac{Y_{\lambda }-\lambda }{\lambda }\right)}^{i}\right)+\lambda -Y_{\lambda }\\ & = & Y_{\lambda }r_{N}\left(\varphi \right)+\left(Y_{\lambda }-\lambda \right)\left(\sum_{i=1}^{N}\frac{{\left(-1\right)}^{i+1}}{i}\;{\left(\frac{Y_{\lambda }-\lambda }{\lambda }\right)}^{i}\right)\\ & & \;\;\;\;\;+\;\lambda \;\left(\sum_{i=1}^{N}\frac{{\left(-1\right)}^{i+1}}{i}\;{\left(\frac{Y_{\lambda }-\lambda }{\lambda }\right)}^{i}\right)+\lambda -Y_{\lambda }\\ & = & Y_{\lambda }r_{N}\left(\varphi \right)+\sum_{i=1}^{N}\frac{{\left(-1\right)}^{i+1}}{i}\;\frac{{\left(Y_{\lambda }-\lambda \right)}^{i+1}}{\lambda^{i}}+\sum_{i=2}^{N}\frac{{\left(-1\right)}^{i+1}}{i}\frac{{\left(Y_{\lambda }-\lambda \right)}^{i}}{\lambda^{i-1}}\\ & = & Y_{\lambda }r_{N}\left(\varphi \right)+\sum_{i=1}^{N}\frac{{\left(-1\right)}^{i+1}}{i}\;\frac{{\left(Y_{\lambda }-\lambda \right)}^{i+1}}{\lambda^{i}}+\sum_{i=1}^{N-1}\frac{{\left(-1\right)}^{i}}{\left(i+1\right)}\frac{{\left(Y_{\lambda }-\lambda \right)}^{i+1}}{\lambda^{i}}\\ & = & Y_{\lambda }r_{N}\left(\varphi \right)+\sum_{i=1}^{N-1}\left(\frac{{\left(-1\right)}^{i+1}}{i}+\frac{{\left(-1\right)}^{i}}{\left(i+1\right)}\right)\frac{{\left(Y_{\lambda }-\lambda \right)}^{i+1}}{\lambda^{i}}+\frac{{\left(-1\right)}^{N+1}}{N}\;\frac{{\left(Y_{\lambda }-\lambda \right)}^{N+1}}{\lambda^{N}}\\ & = & Y_{\lambda }r_{N}\left(\varphi \right)+\sum_{i=1}^{N-1}\frac{{\left(-1\right)}^{i+1}}{i\;(i+1)}\frac{{\left(Y_{\lambda }-\lambda \right)}^{i+1}}{\lambda^{i}}+\frac{{\left(-1\right)}^{N+1}}{N}\;\frac{{\left(Y_{\lambda }-\lambda \right)}^{N+1}}{\lambda^{N}}\;. \end{array}$$

After noting that the only random quantity in (19) is $Y_{\lambda }$, the expected value reads

(20) $$\begin{array}{ccc} \delta^{\left(E\right)}\left(\lambda \right)\;\;=\;\;E\left[F\left(Y_{\lambda }\right)\right] & \;\;\;=\;\;\; & \sum_{i=0}^{N-1}\omega_{i}^{\left(N\right)}\;\frac{\eta_{i+2}\left[Y_{\lambda }\right]}{\lambda^{i+1}}\;\;+\;\;R_{N}\left(\lambda \right)\;, \end{array}$$

with coefficients $\;\omega_{i}^{\left(N\right)}\in Q\;$, i=0,…,N−1, and remainder function $\;R_{N}:R_{++}\to R\;$ given by

(21) $$\omega_{i}^{\left(N\right)}\;=\;\left\{\begin{array}{cc} \frac{{\left(-1\right)}^{i}}{\left(i+1)\;(i+2\right)} & \mathrm{for}\;\;i=0,\ldots ,N-2\\ \frac{{\left(-1\right)}^{i}}{i+1} & \mathrm{for}\;\;i=N-1 \end{array}\right.,\;\;R_{N}\left(\lambda \right)\;=\;E\left[Y_{\lambda }r_{N}\left(\frac{Y_{\lambda }-\lambda }{\lambda }\right)\right],$$

and where

$$\eta_{i+2}\left[Y_{\lambda }\right]\;\;=\;\;E\left[{\left(Y_{\lambda }-\lambda \right)}^{i+2}\right],\;i=0,1,\ldots$$

denote the central moments of order i+2 of the Poisson random variable $Y_{\lambda }$. It is well known (see [13], p. 162) that these moments can be obtained by the recursive formula

(22) $$\eta_{1}\left[Y_{\lambda }\right]=0,\;\;\;\eta_{2}\left[Y_{\lambda }\right]=\lambda ,\;\;\;\eta_{i+2}\left[Y_{\lambda }\right]=\lambda \left(\frac{d\eta_{i+1}\left[Y_{\lambda }\right]}{d\lambda }+\left(i+1\right)\;\eta_{i}\left[Y_{\lambda }\right]\right)\;.$$

After noting that, in (20), only moments $\;\eta_{i+2}\left[Y_{\lambda }\right]\;$ with i≥0 are present and that they are all divided by λ, it is easy to verify that, by applying (22), one obtains the following general algebraic polynomial expression:

(23) $$P_{i}\left(\lambda \right)\;\;:=\;\;\frac{\eta_{i+2}\left[Y_{\lambda }\right]}{\lambda }\;\;=\;\;\sum_{j=0}^{d_{i}}\vartheta_{i}^{\left(j\right)}\lambda^{j}\;,\;\;i=0,1,\ldots \;,$$

where $\vartheta_{i}^{\left(j\right)}$ are all integer coefficients with $\vartheta_{i}^{\left(0\right)}=1\;$ for any i=0,1,…, and where the degrees $\;d_{i}$ of polynomials $P_{i}\left(\lambda \right)$ are given by

(24) $$d_{i}=\left\lfloor \frac{i}{2}\right\rfloor \;\;=\;\;0,0,1,1,2,2,\ldots \;\;\mathrm{for}\;\;\;i\;\;=\;\;0,1,2,3,4,5,\ldots \;,$$

where ⌊·⌋ denotes the floor function. The first 8 polynomials, $P_{i}\left(\lambda \right)$, i=0,…,7, read

$$\begin{array}{ccccccc} P_{0}\left(\lambda \right) & = & 1, & \;\; & P_{1}\left(\lambda \right) & = & 1,\\ P_{2}\left(\lambda \right) & = & 1+3\lambda , & \;\; & P_{3}\left(\lambda \right) & = & 1+10\lambda ,\\ P_{4}\left(\lambda \right) & = & 1+25\lambda +15\lambda^{2}, & \;\; & P_{5}\left(\lambda \right) & = & 1+56\lambda +105\lambda^{2},\\ P_{6}\left(\lambda \right) & = & 1+119\lambda +490\lambda^{2}+105\lambda^{3}, & \;\; & P_{7}\left(\lambda \right) & = & 1+246\lambda +1918\lambda^{2}+1260\lambda^{3}\;. \end{array}$$

By replacing the expressions of $P_{i}\left(\lambda \right)$ given in (23) into (20), one obtains the following general formula:

(25) $$\delta^{\left(E\right)}\left(\lambda \right)\;\;=\;\;E\left[F\left(Y_{\lambda }\right)\right]\;\;=\;\;\sum_{i=0}^{N-1}\left(Q_{i}^{\left(N\right)}\left(\lambda \right)\;\;:=\;\;\sum_{j=0}^{d_{i}}\frac{\psi_{i}^{\left(N,j\right)}}{\lambda^{i-j}}\right)\;\;+\;\;R_{N}\left(\lambda \right)$$

where the coefficients $\;\psi_{i}^{\left(N,j\right)}\in Q\;$ of the rational polynomials $\;Q_{i}^{\left(N\right)}\left(\lambda \right)\;$ in (25) read

$$\psi_{i}^{\left(N,j\right)}\;\;=\;\;\omega_{i}^{\left(N\right)}\vartheta_{i}^{\left(j\right)}\;,\;\;\;i=0,1,\ldots ,N-1,\;\;j=0,1,\ldots ,d_{i},$$

with $\;\omega_{i}^{\left(N\right)}\;$ given in (21) and $\vartheta_{i}^{\left(j\right)}$ defined in (23).

**Figure 3:**
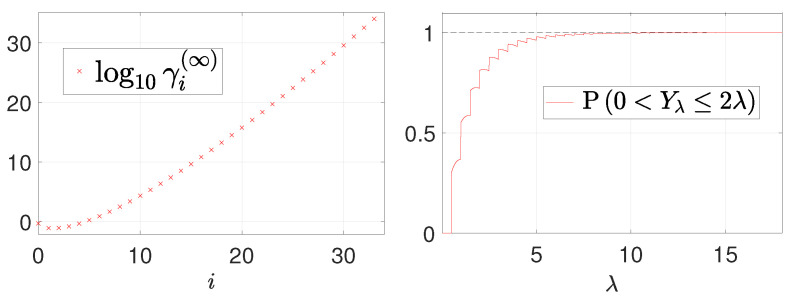
Visual representation of the first 34 terms of the sequence of coefficients $\gamma_{i}^{\left(\infty \right)}$, i=0,1,…, in (26) (**left**) and the behavior of the probability measure defined in (28) as a function of λ (**right**).

After noting that, from (24), it follows that $d_{i}\leq i\;$ for any i=0,1,…, it is a matter of simple algebra to verify that (25) can be equivalently and more compactly rewritten as

(26) $$\delta^{\left(E\right)}\left(\lambda \right)\;\;=\;\;E\left[F\left(Y_{\lambda }\right)\right]\;\;=\;\;\sum_{i=0}^{N-1}\frac{\gamma_{i}^{\left(N\right)}}{\lambda^{i}}\;+\;R_{N}\left(\lambda \right)\;,$$

with $\;\gamma_{i}^{\left(N\right)}\in Q\;$ computable coefficients. In particular, for N=1,…,9, we have

(27) $$\begin{array}{ccc} \delta^{\left(E\right)}\left(\lambda \right) & \;\;\;=\;\;\; & 1+R_{1}\left(\lambda \right)\\ & \;\;\;=\;\;\; & \frac{1}{2}-\frac{1}{2\lambda }+R_{2}\left(\lambda \right)\\ & \;\;\;=\;\;\; & \frac{1}{2}+\frac{5}{6\lambda }+\frac{1}{3\lambda^{2}}+R_{3}\left(\lambda \right)\\ & \;\;\;=\;\;\; & \frac{1}{2}+\frac{1}{12\lambda }-\frac{29}{12\lambda^{2}}-\frac{1}{4\lambda^{3}}+R_{4}\left(\lambda \right)\\ & \;\;\;=\;\;\; & \frac{1}{2}+\frac{1}{12\lambda }+\frac{31}{12\lambda^{2}}+\frac{99}{20\lambda^{3}}+\frac{1}{5\lambda^{4}}+R_{5}\left(\lambda \right)\\ & \;\;\;=\;\;\; & \frac{1}{2}+\frac{1}{12\lambda }+\frac{3}{12\lambda^{2}}-\frac{1003}{60\lambda^{3}}-\frac{93}{10\lambda^{4}}-\frac{1}{6\lambda^{5}}+R_{6}\left(\lambda \right)\\ & \;\;\;=\;\;\; & \frac{1}{2}+\frac{1}{12\lambda }+\frac{1}{12\lambda^{2}}+\frac{797}{60\lambda^{3}}+\frac{687}{10\lambda^{4}}+\frac{713}{42\lambda^{5}}+\frac{1}{7\lambda^{6}}+R_{7}\left(\lambda \right)\\ & \;\;\;=\;\;\; & \frac{1}{2}+\frac{1}{12\lambda }+\frac{1}{12\lambda^{2}}+\frac{19}{120\lambda^{3}}-\frac{3001}{20\lambda^{4}}-\frac{39925}{168\lambda^{5}}-\frac{1721}{56\lambda^{6}}-\frac{1}{8\lambda^{7}}+R_{8}\left(\lambda \right)\\ & \;\;\;=\;\;\; & \frac{1}{2}+\frac{1}{12\lambda }+\frac{1}{12\lambda^{2}}+\frac{19}{120\lambda^{3}}+\frac{1899}{20\lambda^{4}}+\frac{516833}{504\lambda^{5}}+\frac{126829}{168\lambda^{6}}+\frac{4007}{72\lambda^{7}}\\ & & \;\;+\frac{1}{9\lambda^{8}}+R_{9}\left(\lambda \right) \end{array}$$

from which we note how, as the truncation order *N* increases, the coefficients $\gamma_{i}^{\left(N\right)}$ stabilize at some values, which we denote by $\gamma_{i}^{\left(\infty \right)}$. Unfortunately, we are not able to obtain an explicit analytical expression for the sequence of coefficients $\;\gamma_{i}^{\left(\infty \right)}$ (as we are not able to obtain explicit analytic expressions for the coefficients $\vartheta_{i}^{\left(j\right)}$ defining the central moments of a Poisson random variable). By means of the Matlab symbolic toolbox, we were able to compute the first 34 coefficients $\gamma_{i}^{\left(\infty \right)}$, i=0,…,33, shown (in logarithmic scale) in Figure 3 (left). Determining the subsequent coefficients becomes unfeasible due the huge computation time required. Hence, the following short discussion must be regarded as conjectural as it relies on the assumption that the behavior of coefficients $\;\gamma_{i}^{\left(\infty \right)}$, i=34,35,…, can be smoothly extrapolated from the first 34 coefficients shown in Figure 3 (left). These first 34 coefficients indicate that the coefficient sequence is positive and strictly increasing for i≥2. This implies that making the truncation order *N* tend to +∞, the (infinite) weighted geometric series in (26) is divergent for λ≤1. Even without analyzing the case λ>1, we can state that an analytical form for function $\delta^{\left(E\right)}\left(\lambda \right)$ in the low-count Poisson regime is very unlikely to be obtainable as the sum of the series in (26). In fact, there will be, very likely, at least one pixel such that $\lambda_{i}\leq 1$.

We believe that it is worth concluding this section by pointing out the theoretical reason for the non-convergence of the series in (26). Function ln(1+φ) is analytical at φ=0, but its Maclaurin series converges (pointwise to the function) only for φ∈(−1,1]. Hence, as *N* tends to +∞, the Taylor series expansion in (19) converges to the function $F(Y_{\lambda })$ only for $\varphi =\left(Y_{\lambda }-\lambda \right)/\lambda \in \left(-1,1\right]\;⟺\;Y_{\lambda }\in \left(0,2\lambda \right]$. However, $Y_{\lambda }$ in (20) represents a Poisson random variable with parameter λ. Hence, for *N* tending to +∞, the series in (20) converges to the function $\;\delta^{\left(E\right)}\left(\lambda \right)=E\left[F\left(Y_{\lambda }\right)\right]\;$ only if the random variable $\;Y_{\lambda }$ satisfies

(28) $$P\left(0<Y_{\lambda }\leq 2\lambda \right)\;\;=\;\;1\;\;\;⟺\;\;\sum_{i=1}^{\left\lfloor 2\lambda \right\rfloor }P_{\;Y_{\lambda }}\left(i\right)\;\;=\;\;1\;.$$

From Figure 3 (right), where we plot the probability in (28) as a function of λ, one can notice that condition (28) for convergence of the series in (20) is fulfilled asymptotically for λ approaching +∞ but it is not satisfied at all for small λ values.

## 3. A Nearly Exact DP Based on Monte Carlo Simulation

Since it is not possible to derive analytically the expression of function $\;\delta^{\left(E\right)}\left(\lambda \right)$ in (17), the goal in this section is to compute a nearly exact estimate $\;\delta^{\left(NE\right)}\left(\lambda \right)\;$ of function $\;\delta^{\left(E\right)}\left(\lambda \right)\;$ based on a simple Monte Carlo simulation approach analogous to that used at the beginning of Section 2. Based on the expected shape of function $\;\delta^{\left(E\right)}\left(\lambda \right)\;$—see Figure 2(left)—here, we consider a set of 1385 unevenly distributed λ values $\lambda_{i}\in \left[0,250\right]$, namely

$$\lambda_{i}\;\in \;\left\{0,0.01,0.02,\ldots ,5.99,6,6.1,6.2,\ldots ,65.9,66,67,68,\ldots ,249,250\right\}\;.$$

This set comes from the union of three subsets of equally spaced λ values, namely from 0 to 6 with step 0.01, from 6 to 66 with step 0.1, and from 66 to 250 with step 1. For each $\lambda_{i}$, we generate pseudo-randomly a very large number $S=5\times 10^{7}$ of samples $y_{i}^{\left(j\right)}$, j=1,…,S, of the Poisson random variable $Y_{\lambda_{i}}$; then, we compute the associated values $f_{i}^{\left(j\right)}$, j=1,…,S, of the function $F(Y_{\lambda_{i}})$ defined in (16) and, finally, we calculate the sample mean $\;{\hat{\delta }}^{\left(E\right)}\left(\lambda_{i}\right)\;$ and variance $\;v_{i}\;$ of these function values. In formula,

(29) $$\begin{array}{ccc} & y_{i}^{\left(j\right)}\;\;=\;\;\mathrm{Poiss}\left(Y_{\lambda_{i}}\right),\;\;j=1,\ldots ,S\;\;\;\;⟹\;\;\;\;f_{i}^{\left(j\right)}\;\;=\;\;F\left(y_{i}^{\left(j\right)}\right),\;\;j=1,\ldots ,S & \\ & ⟹\;\;\;\;{\hat{\delta }}^{\left(E\right)}\left(\lambda_{i}\right)\;\;=\;\;\frac{1}{S}\sum_{j=1}^{S}f_{i}^{\left(j\right)}\;,\;\;\;v_{i}\;\;=\;\;\frac{1}{S-1}\sum_{j=1}^{S}{\left(f_{i}^{\left(j\right)}-{\hat{\delta }}^{\left(E\right)}\left(\lambda_{i}\right)\right)}^{2}\;. \end{array}$$

**Figure 4:**
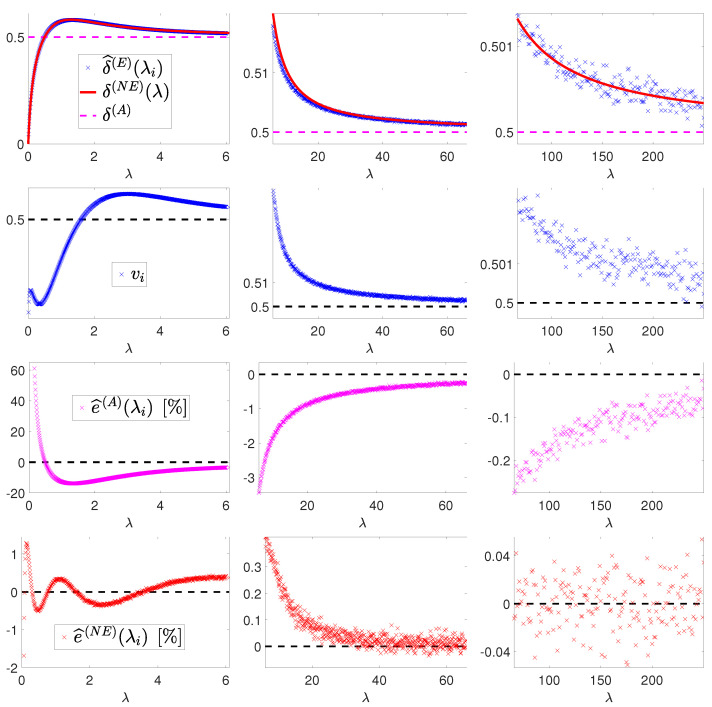
Results of Monte Carlo simulation and weighted least-squares fitting for λ∈[0,6] (**first** column), λ∈[6,66] (**second** column), and λ∈[66,250] (**third** column).

Notation for the sample means come from them representing estimates of the sought theoretical means $\;\delta^{\left(E\right)}\left(\lambda_{i}\right)=E\left[F\left(Y_{\lambda_{i}}\right)\right]\;$, i=1,…,1385. The obtained values $\;\left(\lambda_{i},{\hat{\delta }}^{\left(E\right)}\left(\lambda_{i}\right)\right)\;$ and $\;(\lambda_{i},v_{i})\;$ are shown (blue crosses) in the first and second row of Figure 4, respectively. It is well known that $\;{\hat{\delta }}^{\left(E\right)}\left(\lambda_{i}\right)\;$ and $\;v_{i}\;$ represent unbiased estimators of the mean and standard deviation of the random variable $\;F(Y_{\lambda_{i}})$ and that, according to the central limit theorem, for a very large number *S* of samples (which is definitely our case), the sample mean $\;{\hat{\delta }}^{\left(E\right)}\left(\lambda_{i}\right)\;$ can be regarded as a realization of a Gaussian random variable with the theoretical mean $\;\delta^{\left(E\right)}\left(\lambda_{i}\right)\;$ of the random variable $\;F(Y_{\lambda_{i}})\;$ and the sample variance $\;v_{i}\;$ divided by the number of samples *S*. In formulas,

(30) $${\hat{\delta }}^{\left(E\right)}\left(\lambda_{i}\right)\;\;=\;\;\mathrm{Gauss}\left(\delta^{\left(E\right)}\left(\lambda_{i}\right)\;\;,\;\;\frac{v_{i}}{S}\right)\;.$$

We now want to fit a parametric model f(λ;c), with c the parameter vector, to the obtained Monte-Carlo-simulated data points $\;\left(\lambda_{i},{\hat{\delta }}^{\left(E\right)}\left(\lambda_{i}\right)\right)$, i=1,…,1385. First, in accordance with the trend of these data—see the blue crosses in the first row of Figure 4—and recalling the expected asymptotic behavior of function $\;\delta^{\left(E\right)}\left(\lambda \right)$ for λ approaching +∞—see the discussion in Section 2, particularly the first two terms of the expansion in (27)—we choose a model of the form

(31) $$f\left(\lambda ;c\right)\;\;=\;\;\frac{1}{2}\;+\;\epsilon \left(\lambda ;c\right)\;,$$

with function h exhibiting the following properties:

$$\epsilon \in C^{0}\left(R_{+}\right),\;\;\epsilon \left(0;c\right)\;=\;-\frac{1}{2},\;\;\epsilon \left(\lambda ;c\right)\;∼\;\frac{1}{12\lambda }\;\;\mathrm{for}\;\;\lambda \to +\infty \;.$$

Then, with the aim of achieving a good trade-off between the model’s ability to accurately fit data and the computational efficiency of its evaluation, we choose the following rational form for function ϵ:   

(32) $$\epsilon \left(\lambda ;c\right)\;\;=\;\;\frac{\lambda^{2}+c_{1}\lambda +c_{2}}{12\;\lambda^{3}+c_{3}\lambda^{2}+c_{4}\lambda -2\;c_{2}}\;.$$

Thanks to (30), fitting model *f* in (31) with ϵ as in (32) can be obtained via a Maximum Likelihood (ML) estimation of the parameter vector $c=\left(c_{1},c_{2},c_{3},c_{4}\right)\in R^{4}$. In fact, according to (30), the likelihood reads

(33) $$\begin{array}{ccc} L\left(c\right) & = & \prod_{i=1}^{S}p\left({\hat{\delta }}^{\left(E\right)}\left(\lambda_{i}\right)|c\right)\;\;=\;\;\prod_{i=1}^{S}\;\frac{1}{\sqrt{2\pi \;v_{i}/S}}\;\exp\left(-\frac{1}{2}\frac{{\left({\hat{\delta }}^{\left(E\right)}\left(\lambda_{i}\right)-f\left(\lambda_{i};c\right)\right)}^{2}}{v_{i}/S}\right)\\ & = & \frac{1}{{\left(2\pi /S\right)}^{\frac{S}{2}}\;\prod_{i=1}^{S}\sqrt{v_{i}}}\;\exp\left(-\frac{S}{2}\sum_{i=1}^{S}\frac{{\left({\hat{\delta }}^{\left(E\right)}\left(\lambda_{i}\right)-f\left(\lambda_{i};c\right)\right)}^{2}}{v_{i}}\right)\;, \end{array}$$

and the ML estimate $\;c^{\left(ML\right)}\;$ of c can be computed as follows:

(34) $$c^{\left(ML\right)}\;\in \;\arg\max_{c\;\in \;R^{4}}L\left(c\right)\;=\;\arg\min_{c\;\in \;R^{4}}\left\{-\ln L\left(c\right)\right\}\;=\;\arg\min_{c\;\in \;R^{4}}\sum_{i=1}^{S}w_{i}{\left(d_{i}-h\left(\lambda_{i};c\right)\right)}^{2},$$

where we dropped constants (with respect to the optimization variable c) and defined

$$w_{i}\;\;:=\;\;\frac{1}{v_{i}}\;,\;\;\;d_{i}\;\;:=\;\;{\hat{\delta }}^{\left(E\right)}\left(\lambda_{i}\right)-\frac{1}{2}\;,\;\;\;i=1,\ldots ,S\;.$$

Problem (34) is a nonlinear (in particular, rational) weighted least-squares problem. The cost function is non-convex and local minimizers exist. We compute an estimate $\;\hat{c}\;$ of $\;c^{\left(ML\right)}\;$ by solving (34) via the iterative trust-region algorithm 1000 times starting from 1000 different initial guesses $c^{\left(0\right)}$ randomly sampled from a uniform distribution with support ${\left[-20,20\right]}^{4}$ and then picking up the solution $\;\hat{c}\;$ yielding the minimum cost function value. The obtained parameter estimate is as follows:

(35) $$\hat{c}\;\;=\;\;\left({\hat{c}}_{1},{\hat{c}}_{2},{\hat{c}}_{3},{\hat{c}}_{4}\right)\;\;=\;\;\left(+2.5792,-1.5205,-5.6244,+17.9347\right)\;.$$

We thus define the nearly exact estimate $\;\delta^{\left(NE\right)}\left(\lambda \right)\;$ of the theoretical expected value function $\;\delta^{\left(E\right)}\left(\lambda \right)=E\left[F\left(Y_{\lambda }\right)\right]\;$ as the parametric function f defined in (31), (32) with parameter vector c equal to $\;\hat{c}\;$ given in (35). In formula,

(36) $$\delta^{\left(NE\right)}\left(\lambda \right)\;:=\;f\left(\lambda ;\hat{c}\right)\;=\;\frac{1}{2}\;+\;\epsilon \left(\lambda ;\hat{c}\right)\;=\;\frac{1}{2}\;+\;\frac{\lambda^{2}+2.5792\lambda -1.5205}{12\;\lambda^{3}-5.6244\lambda^{2}+17.9347\lambda +3.0410}\;.$$

In the first row of Figure 4, we plot the constant approximate function $\;\delta^{\left(A\right)}\;$ and the obtained nearly exact function $\;\delta^{\left(NE\right)}\left(\lambda \right)\;$, whereas, in the third and fourth row of Figure 4, we report the errors $\;{\hat{e}}^{\;(A)}\left(\lambda_{i}\right)\;$ and $\;{\hat{e}}^{\;(NE)}\left(\lambda_{i}\right)\;$, respectively. They are defined by

$${\hat{e}}^{\;(X)}\left(\lambda_{i}\right)\;\;=\;\;100\;\times \;\frac{\delta^{\left(X\right)}\left(\lambda_{i}\right)-{\hat{\delta }}^{\;(E)}\left(\lambda_{i}\right)}{{\hat{\delta }}^{\;(E)}\left(\lambda_{i}\right)}\;\;\;\;i=1,2,\ldots ,1385,\;\;X\in \left\{A,NE\right\}\;,$$

and represent the percentage errors associated with using the approximations $\;\delta^{\left(A\right)}\;$ and $\;\delta^{\left(NE\right)}\left(\lambda \right)\;$ with respect to the very accurate Monte Carlo estimates ${\hat{\delta }}^{\;(E)}\left(\lambda_{i}\right)$ of the true underlying expected values $\;\delta^{\left(E\right)}\left(\lambda_{i}\right)=E\left[F\left(Y_{\lambda_{i}}\right)\right]$. One can notice that $\;|{\hat{e}}^{\;(NE)}\left(\lambda_{i}\right)|\;$ is around 20 times smaller than $\;|{\hat{e}}^{\;(A)}\left(\lambda_{i}\right)|\;$ for λ∈[0,6] (first column of Figure 4) and around 10 times less for λ∈[6,250] (second and third column Figure 4). In particular, in the low-count Poisson regime (which we can roughly associate with λ∈[0,6]), the proposed nearly exact estimate of the theoretical expected value function $\;\delta^{\left(E\right)}\left(\lambda \right)\;$ yields a percentage error in the order of 0.5%, whereas the constant approximation used in [5,7] leads to a percentage error in the order of 10%. Such a large error is the reason for the poor performance of the (15) in the low-count regime.

We thus propose the following nearly exact DP (NEDP):

(37) $$\begin{array}{c} \begin{array}{cc} \; & \mathrm{Select}\;\;\;\mu \;=\;\mu^{*}\in R_{+}\;\;\mathrm{such}\;\;\mathrm{that}\;\;\;D\left(\mu^{*};y\right)\;=\;\Delta^{\left(NE\right)}\left(\mu^{*}\right)\;,\\ \; & \mathrm{with}\;\;\Delta^{\left(NE\right)}\left(\mu \right)\;=\sum_{i=1}^{m}\;\left(\delta^{\left(NE\right)}\left({\hat{\lambda }}_{i}\left(\mu \right)\right)\right)\;=\;\frac{m}{2}+\sum_{i=1}^{m}\epsilon \left({\hat{\lambda }}_{i}\left(\mu \right);\hat{c}\right),\\ \; & \;\;\;\;\;\hat{\lambda }\left(\mu \right)\;=\;H\hat{x}\left(\mu \right)+b\;, \end{array} \end{array}$$

with function ϵ and parameter vector $\;\hat{c}\;$ given in (32) and (35), respectively.

## 4. Numerical Solution via ADMM

In the following, we detail how to tackle the minimization problem in (8) and (9) when the regularization parameter μ is automatically selected according to one of the considered versions of the DP, namely the TDP in (13), the ADP in (15), and the NEDP in (37).

In principle, one could set a fine grid of μ-values and compute the solution $\hat{x}\left(\mu \right)$ corresponding to each μ. Then, among the recorded solutions, one could select the one such that the TDP, the ADP, or the NEDP is satisfied. However, this algorithmic scheme, to which we refer as the *a posteriori* optimization procedure, turns out to be particularly costly.

In [6,14], the authors propose to update the regularization parameter according to the ADP along the iterations of the popular Alternating Direction Method of Multipliers (ADMM) [15,16]. Here, we detail the steps of the proposed algorithmic procedure, which can be employed for applying not only the ADP but also the TDP and the NEDP. Finally, we remark that the case of the TDP is only addressed for explanatory purposes and it cannot be performed in practice as $\bar{x}$ is not available.

After introducing the auxiliary variables $\lambda \in R^{m}$, $g\in R^{2n}$, $z\in R^{n}$, problem (8) and (9) can be equivalently written as follows:

(38) $$\begin{array}{c} \begin{array}{cc} \left\{x^{*}\;,\lambda^{*}\;,g^{*},z^{*}\right\}\;\in \; & \underset{x,\lambda ,g,z}{arg min}\left\{\sum_{i=1}^{n}{∥g_{i}∥}_{2}+\;\mu \;\sum_{i=1}^{m}\left(\lambda_{i}\;-\;y_{i}\ln\left(\lambda_{i}\right)\right)\;+\;\iota_{R_{+}^{n}}\;\;\left(z\right)\right\}\\ \; & \mathrm{subject}\;\mathrm{to}\text{:}\;\;\lambda \;=\;Hx+b,\;\;g\;=\;Dx,\;\;z\;=\;x, \end{array} \end{array}$$

where, with a little abuse of notation, we define $g_{i}:=\left({\left(D_{h}x\right)}_{i},{\left(D_{v}x\right)}_{i}\right)\in R^{2}$, for every i=1,…,n.

To solve problem (38), we introduce the augmented Lagrangian function,

(39) $$\begin{array}{c} \begin{array}{cc} L(x,\lambda ,g,\rho_{\lambda },\rho_{g})\;\;=\;\; & \sum_{i=1}^{n}{∥g_{i}∥}_{2}+\;\mu \;\sum_{i=1}^{m}\left(\lambda_{i}\;-\;y_{i}\ln\left(\lambda_{i}\right)\right)\\ \; & -\;\langle \rho_{\lambda },\lambda -\left(Hx+b\right)\rangle \;+\;\frac{\beta_{\lambda }}{2}{∥\lambda -\left(Hx+b\right)∥}_{2}^{2}\\ \; & -\;\langle \rho_{g},g-Dx\rangle \;+\;\frac{\beta_{g}}{2}{∥g-Dx∥}_{2}^{2}\\ \; & -\;\langle \rho_{z},z-x\rangle \;+\;\frac{\beta_{z}}{2}{∥z-x∥}_{2}^{2}+\iota_{R_{+}^{n}}\left(z\right) \end{array} \end{array}$$

where $\rho_{\lambda }\in R^{m}$, $\rho_{g}\in R^{2n}$, $\rho_{z}\in R^{n}$ are the vectors of Lagrange multipliers associated with the linear constraints in (38), while $\beta_{\lambda },\beta_{g},\beta_{z}\in R_{++}$ are the ADMM penalty parameters.

By setting u:=(x;λ;g;z), $\rho =(\rho_{\lambda };\rho_{g};\rho_{z})$, $U:=R^{n}\times R^{m}\times R^{2n}\times R^{n}$, and $R:=R^{m}\times R^{2n}\times R^{n}$, we observe that solving (38) amounts to seeking solutions of the saddle-point problem:   

(40) $$\begin{array}{c} \begin{array}{cc} \; & \mathrm{Find}\;(u^{*},\rho^{*})\in U\;\times \;R\;\;\mathrm{such}\;\mathrm{that}\;\\ \; & L\left(u^{*},\rho \right)\leq L\left(u^{*},\rho^{*}\right)\leq L\left(u,\rho^{*}\right)\;\;\forall \;\left(u,\rho \right)\in U\;\times \;R. \end{array} \end{array}$$

Upon suitable initialization, and for any k≥0, the *k*-th iteration of the ADMM algorithm applied to the solution of (40) with the augmented Lagrangian function L defined in (39) reads

(41) $$\begin{array}{ccc} \lambda^{\left(k+1\right)} & \;\;\in \;\; & \underset{\lambda \in R^{m}}{arg min}L\left(x^{\left(k\right)},\lambda ,g^{\left(k\right)},z^{\left(k\right)},\rho_{\lambda }^{\left(k\right)},\rho_{g}^{\left(k\right)},\rho_{z}^{\left(k\right)}\right)\;, \end{array}$$

(42) $$\begin{array}{ccc} g^{\left(k+1\right)} & \;\;\in \;\; & \underset{g\in R^{2n}}{arg min}L\left(x^{\left(k\right)},\lambda^{\left(k+1\right)},g,z^{\left(k\right)},\rho_{\lambda }^{\left(k\right)},\rho_{g}^{\left(k\right)},\rho_{z}^{\left(k\right)}\right)\;, \end{array}$$

(43) $$\begin{array}{ccc} z^{\left(k+1\right)} & \;\;\in \;\; & \underset{z\in R^{n}}{arg min}L\left(x^{\left(k\right)},\lambda^{\left(k+1\right)},g^{\left(k+1\right)},z,\rho_{\lambda }^{\left(k\right)},\rho_{g}^{\left(k\right)},\rho_{z}^{\left(k\right)}\right)\;, \end{array}$$

(44) $$\begin{array}{ccc} x^{\left(k+1\right)} & \;\;\in \;\; & \underset{x\in R^{n}}{arg min}L\left(x,\lambda^{\left(k+1\right)},g^{\left(k+1\right)},z^{\left(k+1\right)},\rho_{\lambda }^{\left(k\right)},\rho_{g}^{\left(k\right)},\rho_{z}^{\left(k\right)}\right)\;, \end{array}$$

(45) $$\begin{array}{ccc} \rho_{\lambda }^{\left(k+1\right)} & \;\;=\;\; & \rho_{\lambda }^{\left(k\right)}-\beta_{\lambda }\left(\lambda^{\left(k+1\right)}-\left({Hx}^{\left(k+1\right)}+b\right)\right)\;, \end{array}$$

(46) $$\begin{array}{ccc} \rho_{g}^{\left(k+1\right)} & \;\;=\;\; & \rho_{g}^{\left(k\right)}-\beta_{g}\left(g^{\left(k+1\right)}-{Dx}^{\left(k+1\right)}\right)\;, \end{array}$$

(47) $$\begin{array}{ccc} \rho_{z}^{\left(k+1\right)} & \;\;=\;\; & \rho_{z}^{\left(k\right)}-\beta_{z}\left(z^{\left(k+1\right)}-x^{\left(k+1\right)}\right)\;. \end{array}$$

In the following subsections, we discuss the solution of subproblems (41)–(44) for the four primal variables λ, g, z, x. Since the solution of the subproblem for variable λ is the most complicated and requires the application of the DP, it is presented last.

### 4.1. Solving Subproblem for **g**

The subproblem for g in (42) reads

(48) $$\begin{array}{c} \begin{array}{cc} g^{\left(k+1\right)}\;\;\in \;\; & \underset{g\in R^{2n}}{arg min}\left\{\sum_{i=1}^{n}{∥g_{i}∥}_{2}\;-\;\langle \rho_{g}^{\left(k\right)},g-{Dx}^{\left(k\right)}\rangle \;+\;\frac{\beta_{g}}{2}{∥g-{Dx}^{\left(k\right)}∥}_{2}^{2}\right\}\\ \;\;=\;\; & \underset{g\in R^{2n}}{arg min}\left\{\sum_{i=1}^{n}{∥g_{i}∥}_{2}\;+\;\frac{\beta_{g}}{2}{∥g-w^{\left(k\right)}∥}_{2}^{2}\right\},\;\;w^{\left(k\right)}\;=\;{Dx}^{\left(k\right)}+\frac{1}{\beta_{g}}\rho_{g}^{\left(k\right)}\;. \end{array} \end{array}$$

Solving (48) is equivalent to solving the *n* independent two-dimensional minimization problems

$$g_{i}^{\left(k+1\right)}\;=\;\underset{g_{i}\in R^{2}}{arg min}\;\left\{∥g_{i}{∥}_{2}\;+\;\frac{\beta_{g}}{2}{∥g_{i}-w_{i}^{\left(k\right)}∥}_{2}^{2}\right\}\;,\;i=1,\ldots ,n\;,$$

which yields the unique solution

(49) $$g_{i}^{\left(k+1\right)}=w_{i}^{\left(k\right)}\max\left(1-\frac{1}{\beta_{g}{∥w_{i}^{\left(k\right)}∥}_{2}},0\right)\;,\;i=1,\ldots ,n\;.$$

### 4.2. Solving Subproblem for **z**

The subproblem for z in (43) reads

(50) $$\begin{array}{c} \begin{array}{cc} z^{\left(k+1\right)}\;\;\in \;\; & \underset{z\in R^{n}}{arg min}\left\{-\;\langle \rho_{z}^{\left(k\right)},z-x^{\left(k\right)}\rangle \;+\;\frac{\beta_{z}}{2}{∥z-x^{\left(k\right)}∥}_{2}^{2}\;+\iota_{R_{+}^{n}}\left(z\right)\right\}\\ \;\;=\;\; & \underset{z\in R_{+}^{n}}{arg min}\;{∥z-q^{\left(k\right)}∥}_{2}\;,\;\;\;\;q^{\left(k\right)}\;=\;x^{\left(k\right)}+\frac{1}{\beta_{z}}\rho_{z}^{\left(k\right)}\;. \end{array} \end{array}$$

Hence, the solution $z^{\left(k+1\right)}$ is given by the unique Euclidean projection of vector $q^{\left(k\right)}$ defined in (50) onto the (convex) nonnegative orthant and admits the simple closed-form expression

(51) $$z_{i}^{\left(k\right)}\;=\;\max\left(q_{i}^{\left(k\right)}\;,\;0\right),\;i=1,\ldots ,n\;.$$

### 4.3. Solving Subproblem for x

After dropping the constant terms, the x-subproblem in (44) reads:

(52) $$\begin{array}{c} \begin{array}{cc} x^{\left(k+1\right)}\;\in \;\arg\min_{x\in R^{n}}\{\; & -\langle \rho_{\lambda }^{\left(k\right)},\lambda^{\left(k+1\right)}-\left(Hx+b\right)\rangle +\frac{\beta_{\lambda }}{2}{∥\lambda^{\left(k+1\right)}-\left(Hx+b\right)∥}_{2}^{2}\\ \; & -\langle \rho_{g}^{\left(k\right)},g^{\left(k+1\right)}-Dx\rangle +\frac{\beta_{g}}{2}{∥g^{\left(k+1\right)}-Dx∥}_{2}^{2}\\ \; & -\langle \rho_{z}^{\left(k\right)},z^{\left(k+1\right)}-x\rangle +\frac{\beta_{z}}{2}{∥z^{\left(k+1\right)}-x∥}_{2}^{2}\}\;. \end{array} \end{array}$$

By imposing a first-order optimality condition on the quadratic cost function in (52), after simple algebraic manipulations, we obtain the following linear system of equations:

(53) $$\begin{array}{ccc} \left(D^{T}D+\frac{\beta_{\lambda }}{\beta_{g}}H^{T}H+\frac{\beta_{z}}{\beta_{g}}\;I_{n}\right)x & \;=\; & D^{T}\left(g^{\left(k+1\right)}-\frac{1}{\beta_{g}}\rho_{g}^{\left(k\right)}\right)\\ & \;+\; & \frac{\beta_{\lambda }}{\beta_{g}}H^{T}\left(\lambda^{\left(k+1\right)}-b-\frac{1}{\beta_{\lambda }}\rho_{\lambda }^{\left(k\right)}\right)\\ & \;+\; & \frac{\beta_{z}}{\beta_{g}}\left(z^{\left(k+1\right)}-\frac{1}{\beta_{z}}\rho_{z}^{\left(k\right)}\right)\;, \end{array}$$

which is solvable since the coefficient matrix is symmetric positive definite and hence nonsingular. When assuming periodic boundary conditions for x, the blur matrix H is square, i.e., m=n, and, more importantly, $D^{T}D$, $H^{T}H$ and—trivially—I are block circulant matrices with circulant blocks. Hence, the linear system (53) can be solved efficiently by one application of the direct 2D Fast Fourier Transform (FFT) and one application of the inverse 2D FFT, each at a cost of O(nlogn).

### 4.4. Solving Subproblem for λ and Applying the DP

The subproblem for λ in (41) reads

(54) $$\begin{array}{ccc} \lambda^{\left(k+1\right)} & \;\;\;=\;\;\; & \underset{\lambda \;\in \;R^{m}}{arg min}\;\{\;\mu \;\sum_{i=1}^{m}\left(\lambda_{i}\;-\;y_{i}\ln\lambda_{i}\right)-\;\langle \rho_{\lambda }^{\left(k\right)},\lambda -\left({Hx}^{\left(k\right)}+b\right)\rangle \\ & & \;\;\;\;+\;\frac{\beta_{\lambda }}{2}{∥\lambda -\left({Hx}^{\left(k\right)}+b\right)∥}_{2}^{2}\;\;\}\;. \end{array}$$

After manipulating algebraically the last two terms of the cost function in (54), dropping constant terms, and then dividing by the positive scalar $\beta_{\lambda }$, problem (54) can be equivalently rewritten as follows:

(55) $$\begin{array}{ccc} \lambda^{\left(k+1\right)}\left(\gamma \right) & \;\;\;=\;\;\; & \underset{\lambda \;\in \;R^{m}}{arg min}\;\left\{\;\gamma \;\sum_{i=1}^{m}\left(\lambda_{i}\;-\;y_{i}\ln\lambda_{i}\right)\;+\;\frac{1}{2}∥\lambda -v^{\left(k\right)}{∥}_{2}^{2}\;\right\}\;,\\ & & \mathrm{with}\;\;\;\gamma =\frac{\mu }{\beta_{\lambda }}\;,\;\;\;v^{\left(k\right)}=\;{Hx}^{\left(k\right)}+b+\frac{1}{\beta_{\lambda }}\rho_{\lambda }\;. \end{array}$$

In (55), we introduced the explicit dependence of the solution $\lambda^{\left(k+1\right)}$ on the parameter γ, which is the basis of the application of the DP. Notice that the ADMM penalty parameter $\beta_{\lambda }$ is fixed; hence, γ plays the role of the regularization parameter μ in the DP applied to this subproblem. Problem (55) can be further simplified after noting that it can be equivalently rewritten in component-wise (pixel-wise) form as follows:

(56) $$\lambda_{i}^{\left(k+1\right)}\left(\gamma \right)\;\;\in \;\;\underset{\lambda_{i}\;\in \;R_{+}}{arg min}\;\left\{\;\gamma \left(\lambda_{i}\;-\;y_{i}\ln\lambda_{i}\right)\;+\;\frac{1}{2}{\left(\lambda_{i}-v_{i}^{\left(k\right)}\right)}^{2}\;\right\}\;,\;\;\;\;i=1,\ldots ,m\;.$$

It is easy to prove that, for any $\gamma \in R_{+}$ and independently of the constants $y_{i}\in N$ and $v_{i}^{\left(k\right)}\in R$, all the minimization problems in (56) admit a unique solution given by

(57) $$\lambda_{i}^{\left(k+1\right)}\left(\gamma \right)\;\;=\;\;\frac{1}{2}\left(\left(v_{i}^{\left(k\right)}-\gamma \right)\;+\;\sqrt{{\left(v_{i}^{\left(k\right)}-\gamma \right)}^{2}\;+\;4\;y_{i}\gamma }\;\;\right)\;.$$

We now want to apply one among the DP versions—namely, (13), (15), and the proposed (37)—outlined in Section 1 and Section 3 for selecting a value of the free parameter γ in (57). In particular, we select $\gamma =\gamma^{\left(k+1\right)}$ such that $\gamma^{\left(k+1\right)}$ satisfies the discrepancy equation, which, in accordance with the general definition given in (10)–(12), takes here the form

(58) G(γ;y):=D(γ;y)−Δ=0

where the discrepancy function reads

(59) $$D\left(\gamma ;y\right)\;\;=\;\;\sum_{i=1}^{m}D_{i}\left(\gamma ;y_{i}\right)\;\;=\;\;\sum_{i=1}^{m}F\left(\lambda_{i}^{\left(k+1\right)}\left(\gamma \right);y_{i}\right)\;,$$

with function *F* defined in (9), and where the discrepancy value Δ, according to the definitions given in (13), (15) and (37), takes one of the following values/forms:

(60) $$\Delta \;\;=\;\;\left\{\begin{array}{cccc} \Delta^{\left(T\right)}\; & \;\;\;=\;\;\; & \sum_{i=1}^{m}F\left({\left(H\bar{x}+b\right)}_{i}\;;\;y_{i}\right) & \;\mathrm{for}\;\;(13)\;,\;\\ \Delta^{\left(A\right)}\; & \;\;\;=\;\;\; & \frac{m}{2} & \;\mathrm{for}\;\;(15)\;,\;\\ \Delta^{\left(NE\right)}\left(\gamma \right)\; & \;\;\;=\;\;\; & \frac{m}{2}\;+\;\sum_{i=1}^{m}\epsilon \left(\lambda_{i}^{\left(k+1\right)}\left(\gamma \right)\;;\;\hat{c}\right) & \;\mathrm{for}\;\;(37)\;, \end{array}\right.$$

with rational polynomial function ϵ defined in (32) and parameter vector $\;\hat{c}\;$ given in (35). We notice that $\Delta^{\left(T\right)}$ and $\Delta^{\left(A\right)}$ are two positive scalars that can be computed once for all and do not change their values during the ADMM iterations, whereas $\Delta^{\left(NE\right)}\left(\gamma \right)$ is a function of γ, which almost certainly changes its shape along the ADMM iterations (due to function $\lambda_{i}^{\left(k+1\right)}\left(\gamma \right)$ in (57) changing its shape when vector $v^{\left(k\right)}$ in (55) changes).

Summing up, the complete procedure for the DP-based update of the parameter γ and, then, of the variable λ reads as follows:

(61) $$\begin{array}{ccc} v^{\left(k\right)} & \;\;\;=\;\;\; & {Hx}^{\left(k\right)}+b+\frac{1}{\beta_{\lambda }}\rho_{\lambda }\;, \end{array}$$

(62) $$\begin{array}{ccc} \gamma^{\left(k+1\right)} & \;\;\;=\;\;\; & \mathrm{root}\;\;\mathrm{of}\;\;\mathrm{the}\;\;\mathrm{discrepancy}\;\;\mathrm{equation}\;\;\mathrm{in}\;\;(58),(59),(60)\;, \end{array}$$

(63) $$\begin{array}{ccc} \lambda_{i}^{\left(k+1\right)}\left(\gamma^{\left(k+1\right)}\right) & & \mathrm{computed}\;\mathrm{by}\;\;(57)\;,\;\;\mathrm{for}\;\;i=1,\ldots ,m\;. \end{array}$$

Concerning the ADP, in [6], the authors have proven that along the ADMM iterations, the function D(γ;y) is convex and decreasing so that the existence and the uniqueness of the solution of the discrepancy equation in (58) with $\Delta =\Delta^{\left(A\right)}$ is guaranteed. The same result can be immediately extended to the case of the TDP. When considering the NEDP, the functional form of $\Delta^{\left(NE\right)}\left(\gamma \right)$ is such that the above result cannot be straightforwardly applied. However, the following proposition on the existence of a solution for the discrepancy equation (58) with $\Delta =\Delta^{\left(NE\right)}$ holds true.

**Proposition** **1.** *Consider the discrepancy equation in (58)–(60) with $\Delta =\Delta^{\left(NE\right)}\left(\gamma \right)$ and with vector $v^{\left(k\right)}$ and function $\lambda_{i}^{\left(k+1\right)}\left(\gamma \right)$ defined as in (55) and (57), respectively, and let*

(64) $$t^{\left(k\right)}\;\;:=\;\;\max\left\{v^{\left(k\right)},0\right\}\;.$$

*Then, the discrepancy equation admits a solution if the following condition is fulfilled:*

(65) $$\exists \;i:\;\;y_{i}\;\neq \;0\;\;\;\;\;\wedge \;\;\;\;T\left(t^{\left(k\right)},y\right)\;\;:=\;\;\sum_{i=1}^{m}T\left(t_{i}^{\left(k\right)},y_{i}\right)\;\;\geq \;\;\frac{m}{2}\;,$$

*where function $\;T\;:\;\;R_{+}\;\times \;N\to R\;$ is defined by*

(66) $$T\left(t,y\right)\;\;=\;\;F\left(t;y\right)-\epsilon \left(t\;;\;\hat{c}\right)\;,$$

*with function F, function ϵ, and parameter vector $\;\hat{c}\;$ given in *(9)*, *(32)*, and *(35)*, respectively.*

**Proof.**  Since functions *F* in (9), ϵ in (32), and $\lambda_{i}^{\left(k+1\right)}$ in (57) are all continuous, then the function *G* defined in (58)–(60) with $\Delta =\Delta^{\left(NE\right)}\left(\gamma \right)$ is continuous in the variable γ on its entire domain $\gamma \in R_{+}$, for any $y\in N^{m}$ and any $v^{\left(k\right)}\in R^{m}$.

Then, it is easy to prove that function $\lambda_{i}^{\left(k+1\right)}\left(\gamma \right)$ in (57) satisfies

(67) $$\lambda_{i}^{\left(k+1\right)}\left(0\right)\;\;=\;\;\max\left\{v_{i}^{\left(k\right)},0\right\}\;\;=\;\;t_{i}^{\left(k\right)}\;,\;\;\;\;\lim_{\gamma \to +\infty }\lambda_{i}^{\left(k+1\right)}\left(\gamma \right)\;\;=\;\;y_{i}\;,$$

with vector $\;t^{\left(k\right)}$ defined in (64).

It thus follows from (67) and from the definition of functions D in (59) and $\Delta^{\left(NE\right)}$ in (60) that

(68) $$\begin{array}{ccc} G(0;y) & \;\;\;=\;\;\; & D\left(0;y\right)-\Delta^{\left(NE\right)}\left(0\right)\;\;=\;\;\sum_{i=1}^{m}F\left(\lambda_{i}^{\left(k+1\right)}\left(0\right);y_{i}\right)-\frac{m}{2}-\sum_{i=1}^{m}\epsilon \left(\lambda_{i}^{\left(k+1\right)}\left(0\right)\;;\;\hat{c}\right)\\ & \;\;\;=\;\;\; & \sum_{i=1}^{m}\left(F\left(t_{i}^{\left(k\right)};y_{i}\right)-\epsilon \left(t_{i}^{\left(k\right)}\;;\;\hat{c}\right)\right)-\frac{m}{2}\;\;=\;\;T\left(t^{\left(k\right)},y\right)-\frac{m}{2}\;, \end{array}$$

and that

limγ→+∞G(γ;y)=limγ→+∞D(γ;y)−Δ(NE)(γ)=limγ→+∞∑i=1mFλi(k+1)(γ);yi−m2−∑i=1mϵλi(k+1)(γ);c^(69)=∑i=1mF(yi;yi)−∑i=1m12+ϵyi;c^(70)=−∑i=1mfyi;c^<0if∃i:yi≠0,

where function T in (68) is defined in (65), cancelling the first summation in (69) comes from F(y;y)=0 for any $y\in R_{+}$ (see the definition of function *F* in (9), where ylny=0 for y=0) and (70) comes from the definition of function *f* in (36).

From (70) and the continuity of function G(γ;y), we can conclude that, for any y≠0, the discrepancy equation G(γ;y)=0 admits a solution if G(0;y)≥0. It thus follows from (68) that the sufficient condition in (64)–(66) holds true.   □

In Algorithm 1, we outline the general ADMM-based scheme used for solving image restoration variational models of the TV-KL form in (8) and (9) with automatic update/selection of the regularization parameter μ according to one of the considered versions of the DP. We refer to the general scheme as DP-ADMM, whereas the specific schemes using one among the DP versions TDP, ADP, and NEDP will be named TDP-ADMM, ADP-ADMM, and NEDP-ADMM, respectively. Notice that the γ-update at step 3 can be performed by means of a derivative-free approach, such as bisection or the secant method.

**Algorithm 1:** General DP-ADMM approach for image restoration variational models of the TV-KL form in (8) and (9) and automatic selection of μ via DP.**inputs**: observed degraded image $\;y\in N^{m}$, emission background $b\in R_{+}^{m}$, blur and regularization operators $H\in R^{m\times n}$, $D\in R^{2n\times n}$**output**: estimated restored image $\;\hat{x}\in R^{n}$    1. **initialise:** set $\;x^{\left(0\right)}=y$  2. **for**
*k = 0, 1, 2, … until convergence*
**do**:    3.    · compute $\;\gamma^{\left(k+1\right)}=\mu^{\left(k+1\right)}/\beta_{\lambda }\;\;\;\;$by (61) and (62)  4.    · compute $\;\lambda^{\left(k+1\right)}$by (63)  5.    · compute $\;\;g^{\left(k+1\right)}$by (49)  6.    · compute $\;\;z^{\left(k+1\right)}$by (51)  7.    · compute $\;\;x^{\left(k+1\right)}$by (53)  8.    · compute $\;\;\rho_{\lambda }^{\left(k+1\right)},\rho_{g}^{\left(k+1\right)},\rho_{z}^{\left(k+1\right)}$by (45)–(47)  9. **end for**  10. $\hat{x}=x^{\left(k+1\right)}$


## 5. Numerical Results

In this section, we evaluate the performance of the proposed NEDP in (37) for the automatic selection of the regularization parameter μ in image restoration variational models of the TV-KL form in (8) and (9).

Our approach is compared with the TDP and the ADP in (13) and (15), respectively. For each criterion, we perform the ADMM-based scheme outlined in Algorithm 1. As recalled in Section 4, the μ-selection problem along the ADMM iterations always admits a unique solution under the adoption of the ADP and TDP. Concerning the NEDP-ADMM, at the generic iteration *k* of Algorithm 1, the regularization parameter μ is updated provided that the condition stated in Proposition 1 is satisfied. If this is not the case, the parameter update is not performed and $\mu^{\left(k\right)}=\mu^{\left(k-1\right)}$.

The numerical tests have been designed with the following twofold aim:

(i) to prove that the proposed NEDP criterion is capable of selecting optimal μ values returning high-quality restoration results and, in particular, that it outperforms the classical ADP criterion in the low-count Poisson regime;
(ii) to prove that the proposed NEDP-ADMM scheme outlined in Algorithm 1 is capable of automatically selecting such optimal μ values in a robust (and efficient) manner.

More specifically, the latter point will be proven by showing that the iterated and the *a posteriori* version of our approach behave similarly in terms of μ-selection.

The μ-values selected by the TDP, the ADP, and the NEDP applied *a posteriori* will be denoted by $\mu^{\left(T\right)},\mu^{\left(A\right)},\mu^{\left(NE\right)}$, respectively, while the output μ-value of the ADP-ADMM and of the NEDP-ADMM scheme will be denoted by ${\hat{\mu }}^{\left(A\right)}$ and ${\hat{\mu }}^{\left(NE\right)}$, respectively.

The quality of the restorations $\hat{x}$ with respect to the original uncorrupted image $\bar{x}$ will be assessed by means of two scalar measures, namely the Structural Similarity Index (SSIM) [17] and the Improved-Signal-to-Noise Ratio (ISNR), defined by

$$\mathrm{ISNR}\left(\hat{x},\bar{x}\right)\;\;=\;\;10\log_{10}\frac{∥\bar{x}{-b∥}_{2}^{2}}{∥\bar{x}-\hat{x}{∥}_{2}^{2}}\;.$$

For all tests, the iterations of the ADMM-based scheme in Algorithm 1 are stopped as soon as

$$\epsilon_{x}^{\left(k\right)}=\frac{∥x^{\left(k\right)}-x^{\left(k-1\right)}{∥}_{2}}{∥x^{\left(k-1\right)}{∥}_{2}}<10^{-5}\;,\;k\in N\setminus \left\{0\right\}\;,$$

and the ADMM penalty parameters $\;\beta_{\lambda },\beta_{g},\beta_{z}$ are set manually so as to achieve fast convergence. More precisely, in each test, the same triplet $\left(\beta_{\lambda },\beta_{g},\beta_{z}\right)$ of ADMM penalty parameters is used for the three compared discrepancy principles TDP, ADP, and NEDP, with $\beta_{\lambda },\beta_{g},\beta_{z}\in \left[0.5,2\right]$.

**Figure 5:**
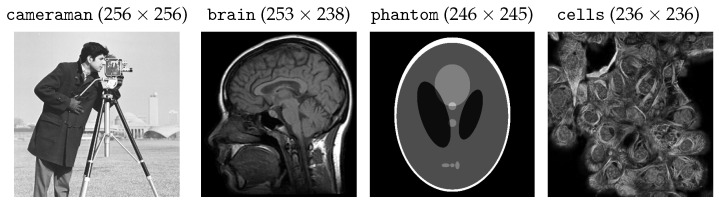
Grayscale test images considered for the numerical experiments.

We consider the four test images, each with pixel values between 0 and 1, shown in Figure 5. The acquisition process (1) has been simulated as follows. First, the original image is multiplied by a factor κ∈N∖{0} representing the maximum emitted photon count, i.e., the maximum expected value of the number of photons emitted by the scene and hitting the image domain. Clearly, the lower κ, the lower the SNR of the observed noisy image and the more difficult the image restoration problem. For each image, several values κ ranging from 3 to 1000 have been considered. Then, the resulting images have been corrupted by space-invariant Gaussian blur, with a blur kernel generated by the Matlab routine fspecial, which is characterized by two parameters, namely the band parameter, representing the side length (in pixels) of the square support of the kernel, and sigma, which is the standard deviation (in pixels) of the isotropic bivariate Gaussian distribution defining the kernel in the continuous setting. We consider two different blur levels characterized by the parameters band = 5, sigma = 1 and band = 13, sigma = 3. The blurred noiseless image λ=Hx+b is then generated by adding to the blurred image a constant emission background b of value $2\times 10^{-3}$. The observed image y=Poiss(λ) is finally obtained by pseudo-randomly generating an *m*-variate independent Poisson realization with mean vector λ.

**Figure 6:**
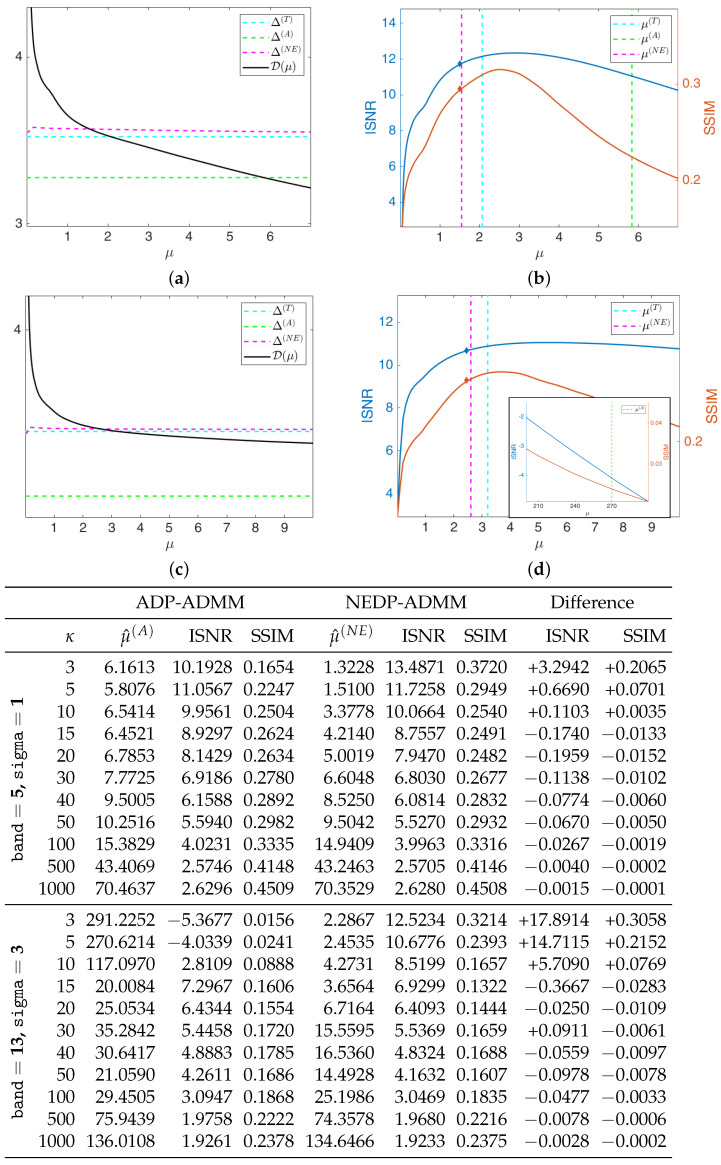
Test image cameraman. **Top**: discrepancy curve divided by $10^{4}$ (**a**,**c**) and ISNR/SSIM values (**b**,**d**) achieved for different μ-values with κ=5 and Gaussian blur with parameters band = 5, sigma = 1 (**first** row) and band = 13, sigma = 3 (**second** row). **Bottom**: output μ-values and ISNR/SSIM values obtained by the ADP-ADMM and the NEDP-ADMM for the two blur levels considered and different photon counts κ.

The black solid curves plotted in Figure 6a,c represent the function D(μ;y) as defined in (11) and (12) for the first test image cameraman and κ=5, for the less severe (first row) and more severe (second row) blur level. They have been computed by solving the TV-KL model in (8) for a fine grid of different μ-values, and then calculating D(μ;y) for each μ. The horizontal dashed cyan and green lines represent the constant discrepancy values $\Delta^{\left(T\right)}$ and $\Delta^{\left(A\right)}$ used in (13) and (15), respectively, while the dashed magenta curve represents the discrepancy value function $\Delta^{\left(NE\right)}\left(\mu \right)$ defined in (37). We remark that $\Delta^{\left(NE\right)}\left(\mu \right)$ has been obtained in the same way as D(μ;y), i.e., by computing the expression in (37) for each μ of the selected fine grid. One can clearly observe that the intersection points between the curve $\Delta^{\left(NE\right)}\left(\mu \right)$ and the function D(μ;y) and between the line representing $\Delta^{\left(T\right)}$ and D(μ;y) are very close, and both at a significant distance from the intersection point detected by $\Delta^{\left(A\right)}$.

In Figure 6b,d, we show the INSR and SSIM values achieved for different μ-values with κ=5. The vertical cyan, green, and magenta lines correspond to the μ-values detected by the intersection of D(μ;y) and $\Delta^{\left(T\right)}$, $\Delta^{\left(A\right)}$, $\Delta^{\left(NE\right)}\left(\mu \right)$, respectively. As a reflection of the behavior of the discrepancy function and of the three curves, the ISNR/SSIM corresponding to $\mu^{\left(T\right)}$ and $\mu^{\left(NE\right)}$ are very close to each other and almost reach the maximum of the two curves. We also highlight that, when considering the more severe blur case, the ADP selects a very large μ-value, which returns very low ISNR and SSIM values—see the thumbnail image in the right-hand corner of Figure 6d.

We are also interested in verifying that the proposed NEDP-ADMM scheme outlined in Algorithm 1 succeeds in automatically selecting such optimal μ in a robust and efficient way: the blue and red markers in Figure 6b,d represent the final ISNR and SSIM values, respectively, of the image restored via NEDP-ADMM. Clearly, the markers are plotted in correspondence of ${\hat{\mu }}^{\left(NE\right)}$, which is, as we recall, the output μ-value of the iterative scheme NEDP-ADMM; one can clearly observe that ${\hat{\mu }}^{\left(NE\right)}$ is very close to the optimal $\mu^{\left(NE\right)}$ detected *a posteriori* by the NEDP.

As a further analysis, at the bottom of Figure 6, we report the output μ-values, the ISNR and the SSIM values for the two blur levels, and the 11 κ-values considered to be obtained by the ADP-ADMM (first column) and the NEDP-ADMM (second column). To facilitate the comparison, we also report in blue/red the increments/decrements of the ISNR and SSIM achieved by our method with respect to the approximate criterion. Notice that the NEDP outperforms the ADP both in terms of ISNR and SSIM for the low-count acquisitions. However, when the κ increases, the two methods behave very similarly, with the ISNR and SSIM values obtained by the ADP-ADMM being slightly larger than those obtained by the NEDP-ADMM. In accordance with this analysis, the output ${\hat{\mu }}^{\left(A\right)}$ and ${\hat{\mu }}^{\left(NE\right)}$ are significantly different in low-count regimes, similar in mid-count regimes, and particularly close in high-count regimes. Notice that this behavior can be easily explained in light of the analysis carried out in Section 2, where we have shown that the approximation provided by $\Delta^{\left(A\right)}$ becomes more and more accurate as the number of pixels with large values increases.

**Figure 7:**
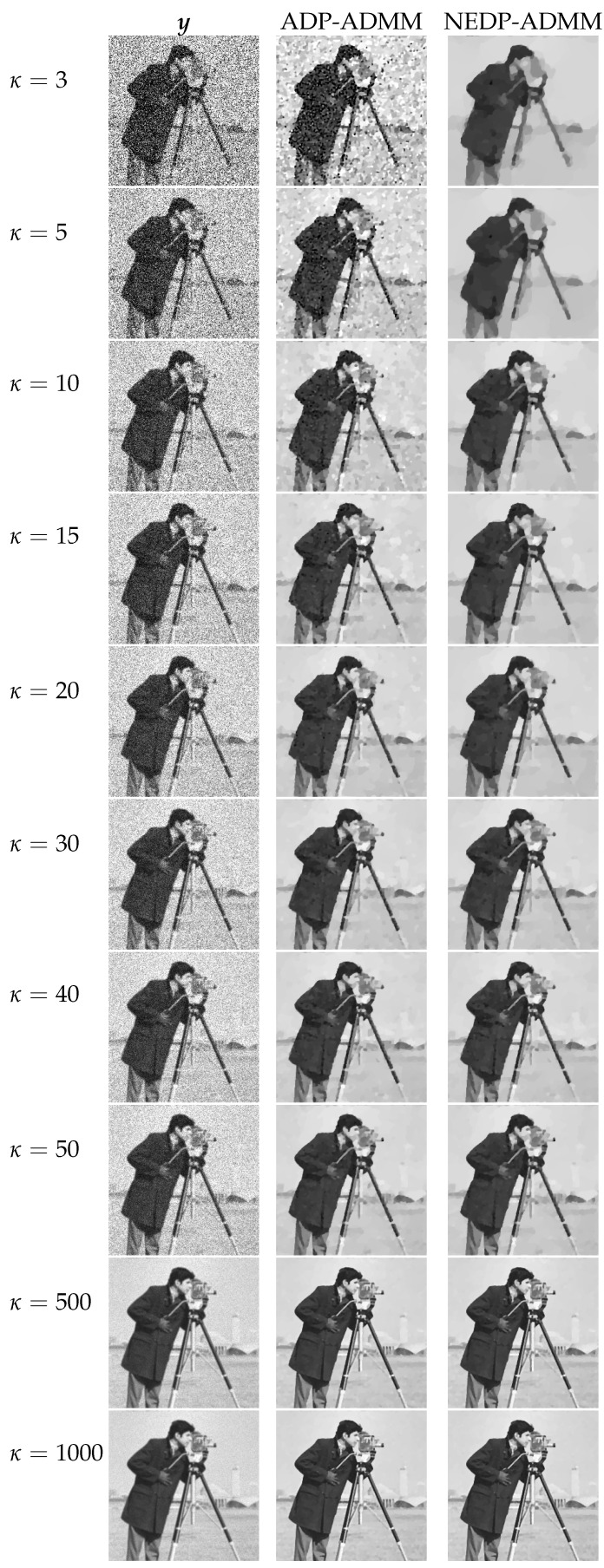
Test image cameraman. **Left** column: observed data y corrupted by Gaussian blur with parameters band = 5, sigma = 1 and Poisson noise with different κ-values ranging from 3 to 1000. **Middle** column: restorations by ADP-ADMM. **Right** column: restorations by NEDP-ADMM.

**Figure 8:**
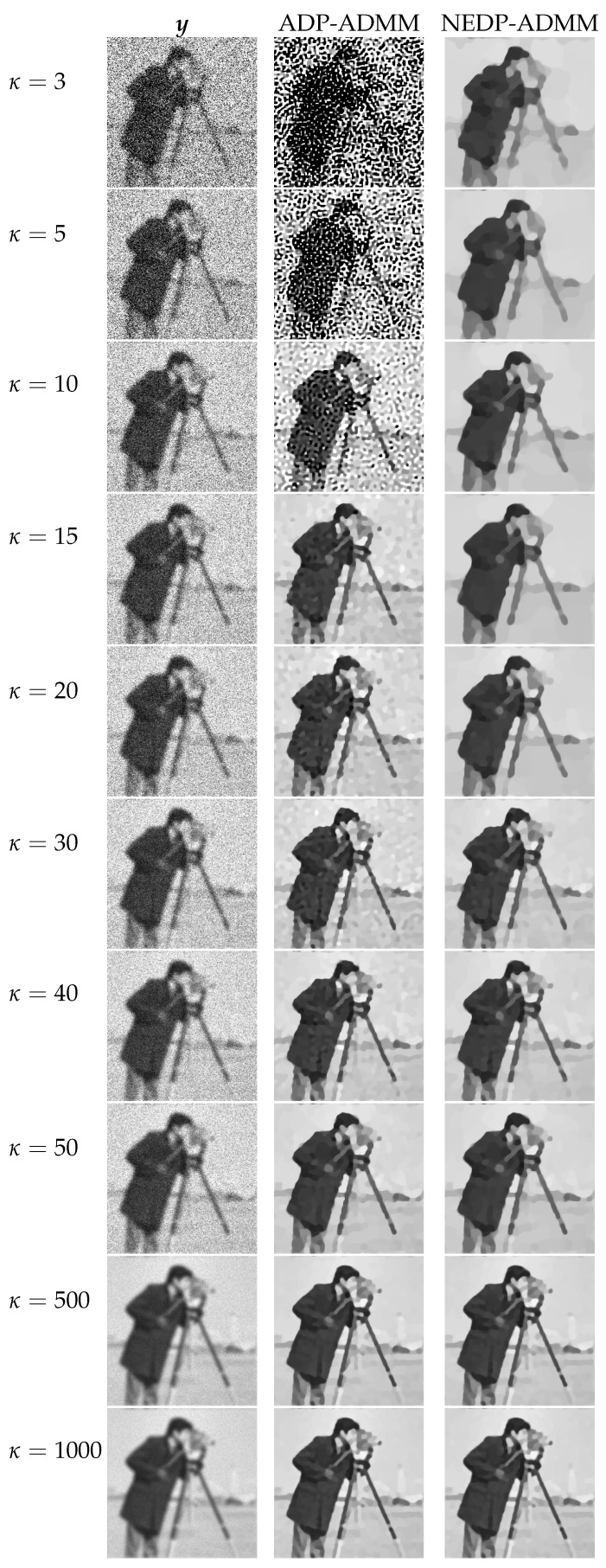
Test image cameraman. **Left** column: observed data y corrupted by Gaussian blur with parameters band = 13, sigma = 3 and Poisson noise with different κ-values ranging from 3 to 1000. **Middle** column: restorations by ADP-ADMM. **Right** column: restorations by NEDP-ADMM.

For a visual comparison, in Figure 7 and Figure 8, we show the observed images (left column), the restorations via ADP-ADMM (middle column) and via NEDP-ADMM (right column) for the less and more severe blur level, respectively, and when different photon count regimes, ranging from very low to very high, are considered. As already observed from the ISNR and SSIM values reported at the bottom of Figure 6, we notice that for low-count acquisitions, the μ-value selected by the ADP does not allow for a proper regularization, so that NEDP-ADMM clearly outperforms the competitor. However, starting from κ=15—for the first blur level—and from κ=40—for the second blur level—the two approaches return similar output images.

**Figure 9:**
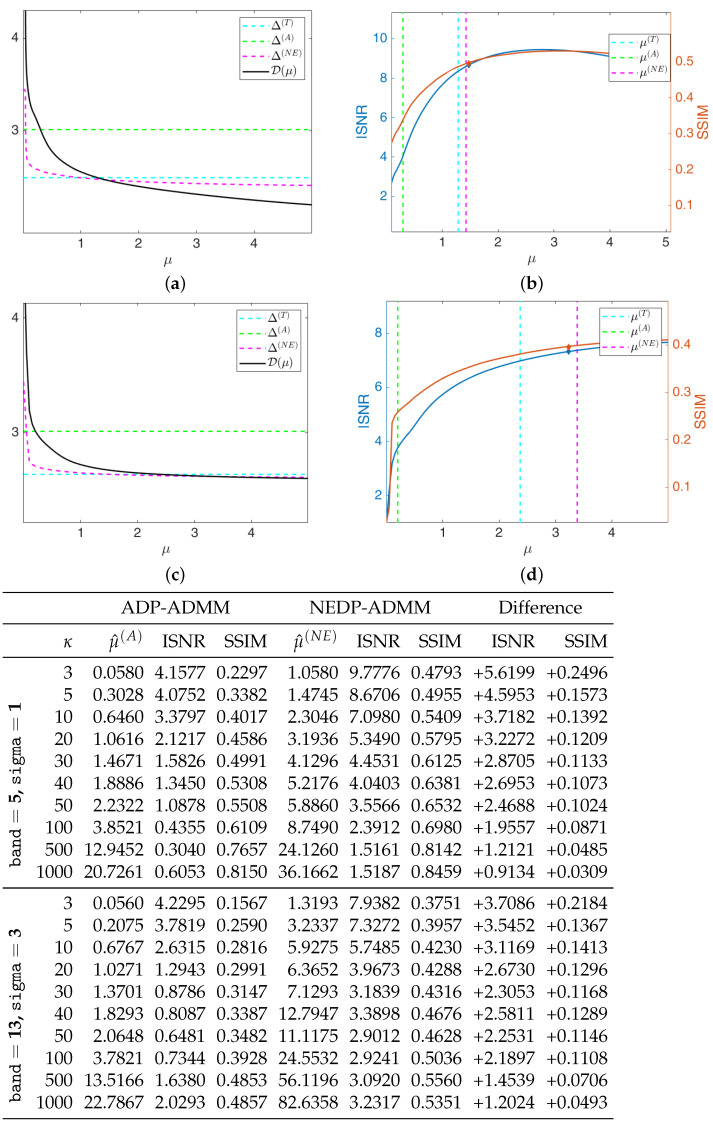
Test image brain. **Top**: discrepancy curve divided by $10^{4}$ (**a**,**c**) and ISNR/SSIM values (**b**,**d**) achieved for different μ-values with κ=5 and Gaussian blur with parameters band = 5, sigma = 1 (**first** row) and band = 13, sigma = 3 (**second** row). **Bottom**: output μ-values and ISNR/SSIM values obtained by the ADP-ADMM and the NEDP-ADMM for the two blur levels considered and different photon counts κ.

For the second test image, brain, we report in Figure 9 the behavior of the discrepancy function D(μ;y) and of the ISNR/SSIM curves obtained by applying the TDP, the ADP, and the NEDP, for κ=5 and for the two considered blur levels. In addition, in this case, the NEDP and the TDP behave similarly and they almost achieve the maximum of the ISNR and of the SSIM curves. In contrast, $\mu^{\left(A\right)}$ appears to be largely underestimated with respect to the optimal μ—which can be intended as the one maximizing either the ISNR or the SSIM. As for the first test image, the blue and red markers, indicating the output ISNR and SSIM, respectively, of the iterated version of our approach, are very close to the ones achieved by applying the NEDP *a posteriori*, suggesting that also ${\hat{\mu }}^{\left(NE\right)}$ and $\mu^{\left(NE\right)}$ are very close.

From the table reported at the bottom of Figure 9, we observe that the proposed μ-selection criterion, for every κ, returns restored images outperforming the ones achieved via the ADP both in terms of ISNR and SSIM. The poor behavior of the ADP can be related to the nature of the processed image, which, either for the low-count or high-count acquisitions, presents few pixels with large values so that the approximation in (15) is particularly inaccurate. As a signal of this, note that the output ${\hat{\mu }}^{\left(A\right)}$ is always smaller—or significantly smaller—than ${\hat{\mu }}^{\left(NE\right)}$.

**Figure 10:**
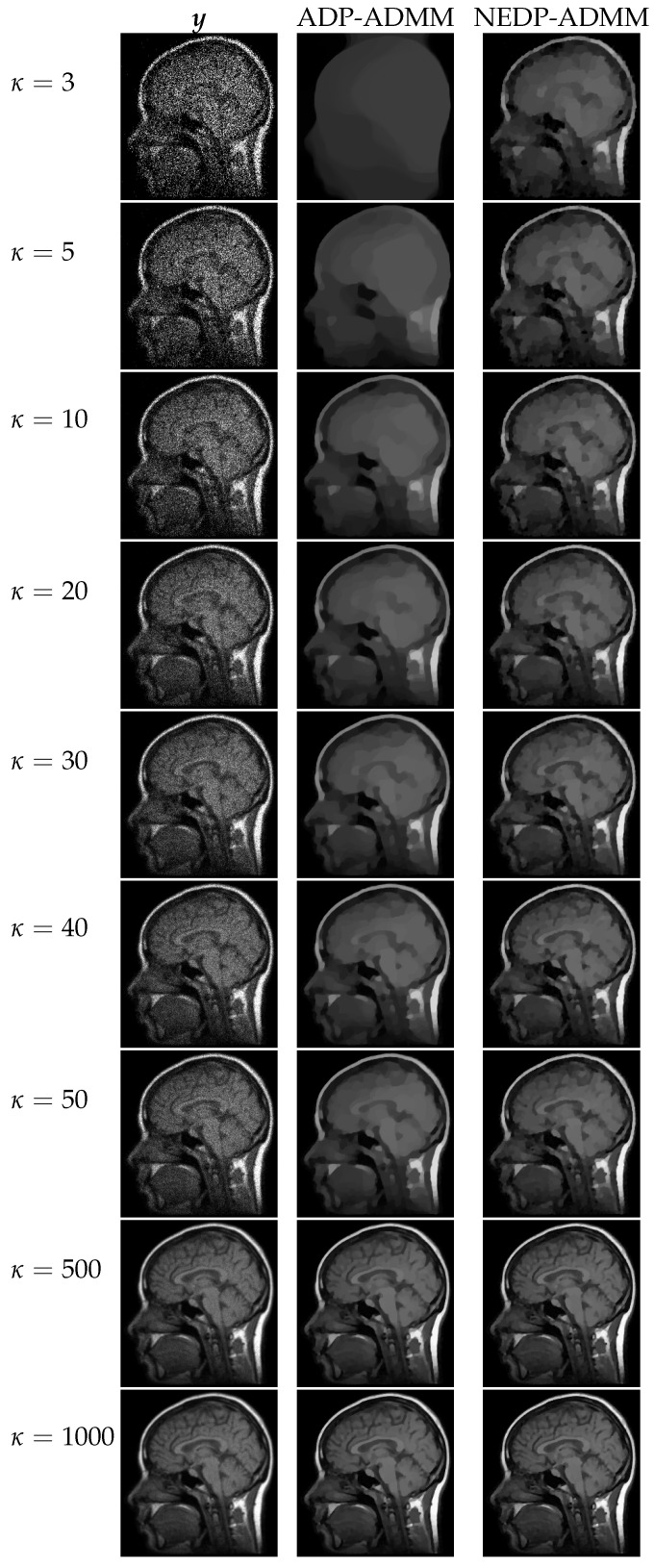
Test image brain. **Left** column: observed data y corrupted by Gaussian blur with parameters band = 5, sigma = 1 and Poisson noise with different κ-values ranging from 3 to 1000. **Middle** column: restorations by ADP-ADMM. **Right** column: restorations by NEDP-ADMM.

**Figure 11:**
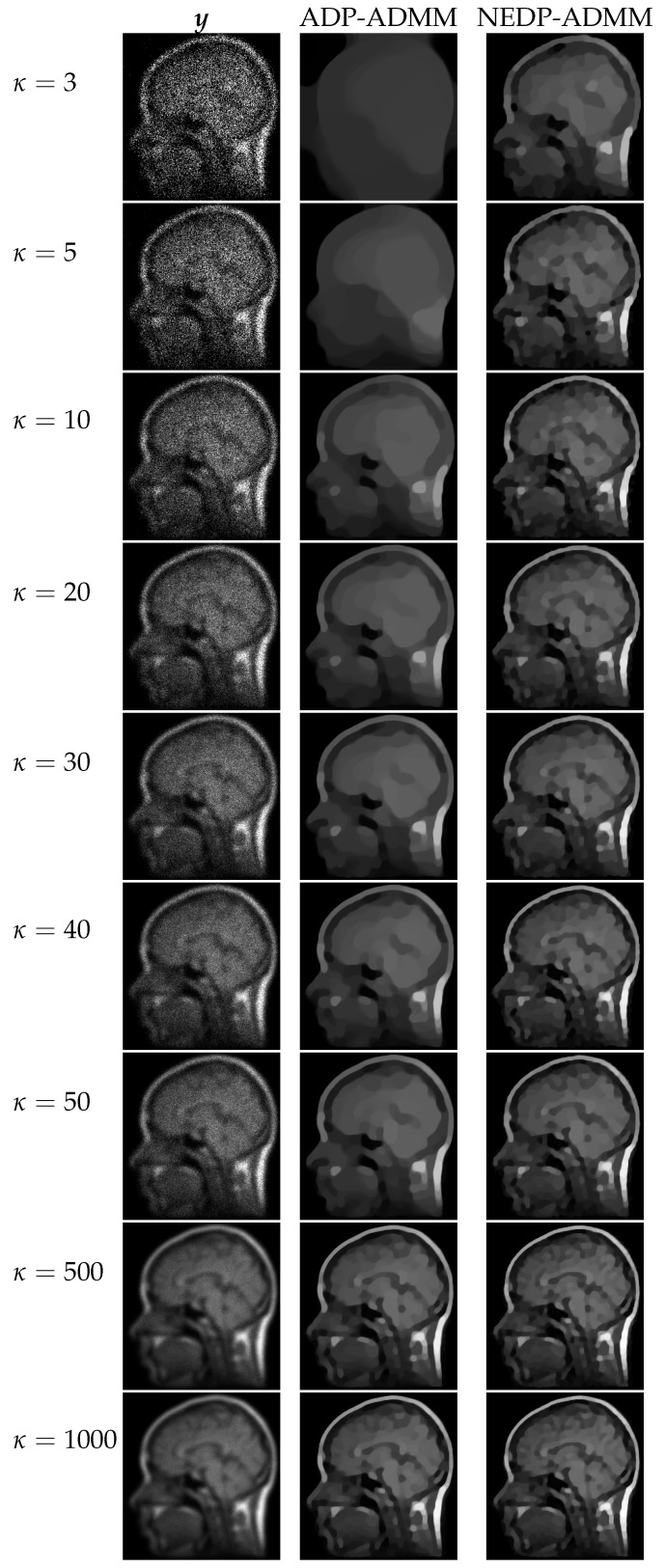
Test image brain. **Left** column: observed data y corrupted by Gaussian blur with parameters band = 13, sigma = 3 and Poisson noise with different κ-values ranging from 3 to 1000. **Middle** column: restorations by ADP-ADMM. **Right** column: restorations by NEDP-ADMM.

The restored images in Figure 10 and Figure 11 reflect the values recorded in the table as, for the two considered blur levels, the output of the NEDP-ADDM appears to be remarkably sharper than the final restoration by ADP-ADMM, especially in low- and mid-count regimes.

**Figure 12:**
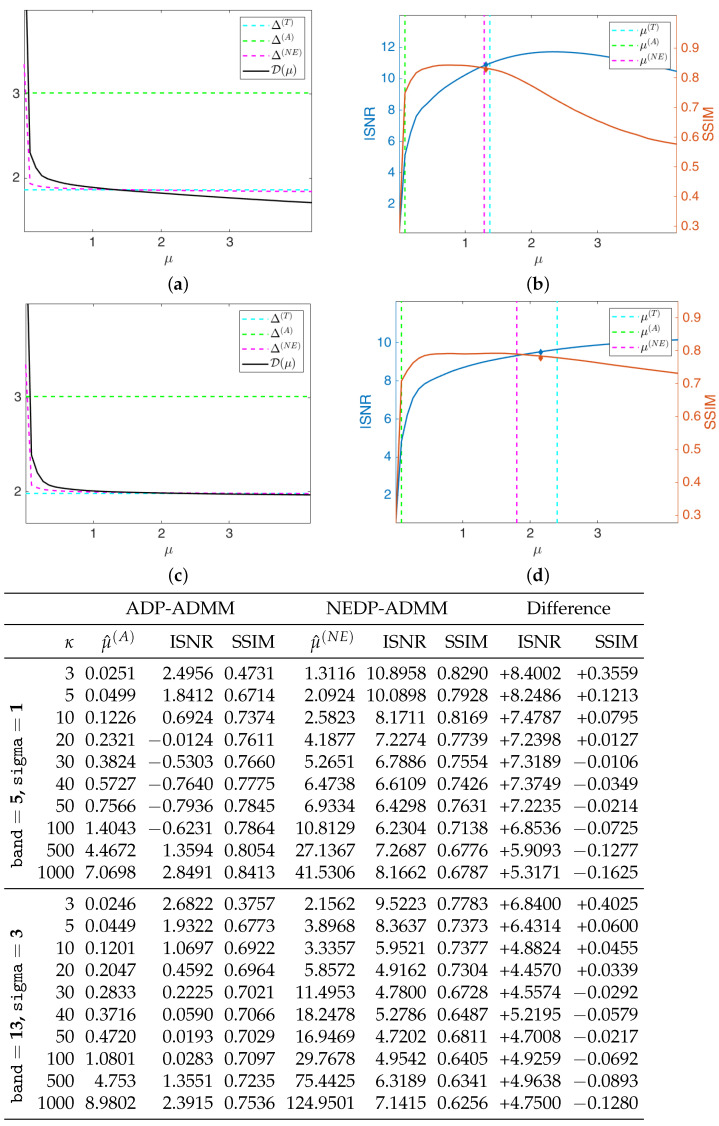
Test image phantom. **Top**: discrepancy curve divided by $10^{4}$ (**a**,**c**) and ISNR/SSIM values (**b**,**d**) achieved for different μ-values with κ=3 and Gaussian blur with parameters band = 5, sigma = 1 (**first** row) and band = 13, sigma = 3 (**second** row). **Bottom**: output μ-values and ISNR/SSIM values obtained by the ADP-ADMM and the NEDP-ADMM for the two blur levels considered and different photon counts κ.

In Figure 12, for the test image phantom, we report the curve of the discrepancy function D(μ;y) obtained *a posteriori*, as well as the curves of the ISNR and of the SSIM for κ=3 and the two blur levels considered. As for the test image brain, in this case, the ADP also selects a μ-value that is far from the optimal one, either if measured in terms of ISNR or SSIM. On the other hand, $\mu^{\left(T\right)}$ and $\mu^{\left(NE\right)}$ are very close and, in particular, one can notice that the output of the NEDP-ADMM represents the optimal compromise in terms of ISNR and SSIM. Notice also that, when considering the larger blur level, the output value ${\hat{\mu }}^{\left(NE\right)}$ of the NEDP-ADMM, detected by the red and blue markers, is larger than the one selected by the *a posteriori* version of the NEDP. However, the difference in terms of ISNR and SSIM is not particularly significant. This behavior is due to the use of different penalty parameters $\beta_{\lambda },\beta_{g},\beta_{z}$ in the ADMM for the *a posteriori* and the iterated version of our approach. In fact, when considering a large blur level, the convergence of the ADMM is particularly slow and can be achieved upon suitable selection of the penalty parameters, whose values may not coincide in the two scenarios addressed.

The mismatch observed in Figure 12 between the curves of the ISNR and of the SSIM is reflected in the values reported in the bottom part of the figure, whence we can conclude that the NEDP-ADMM outperforms the ADP-ADMM in terms of ISNR for each κ-value, while the ADP-ADMM returns slightly better results in terms of SSIM for high-count acquisitions. As for the test image brain, in this case, the output ${\hat{\mu }}^{\left(A\right)}$ also appears to be significantly small. Once again, this behavior can be related to the considered image, which is mostly characterized by pixels with very small values.

**Figure 13:**
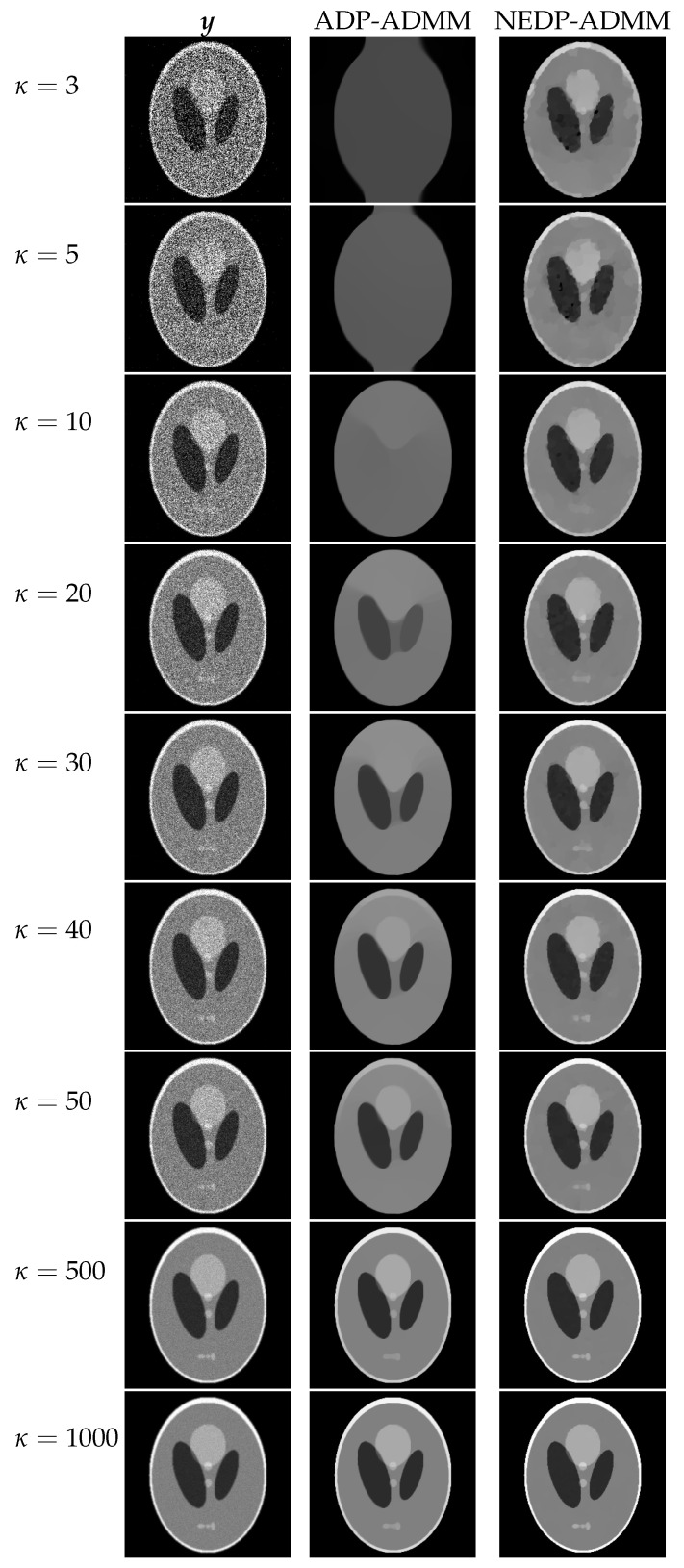
Test image phantom. **Left** column: observed data y corrupted by Gaussian blur with parameters band = 5, sigma = 1 and Poisson noise with different κ-values ranging from 3 to 1000. **Middle** column: restorations by ADP-ADMM. **Right** column: restorations by NEDP-ADMM.

**Figure 14:**
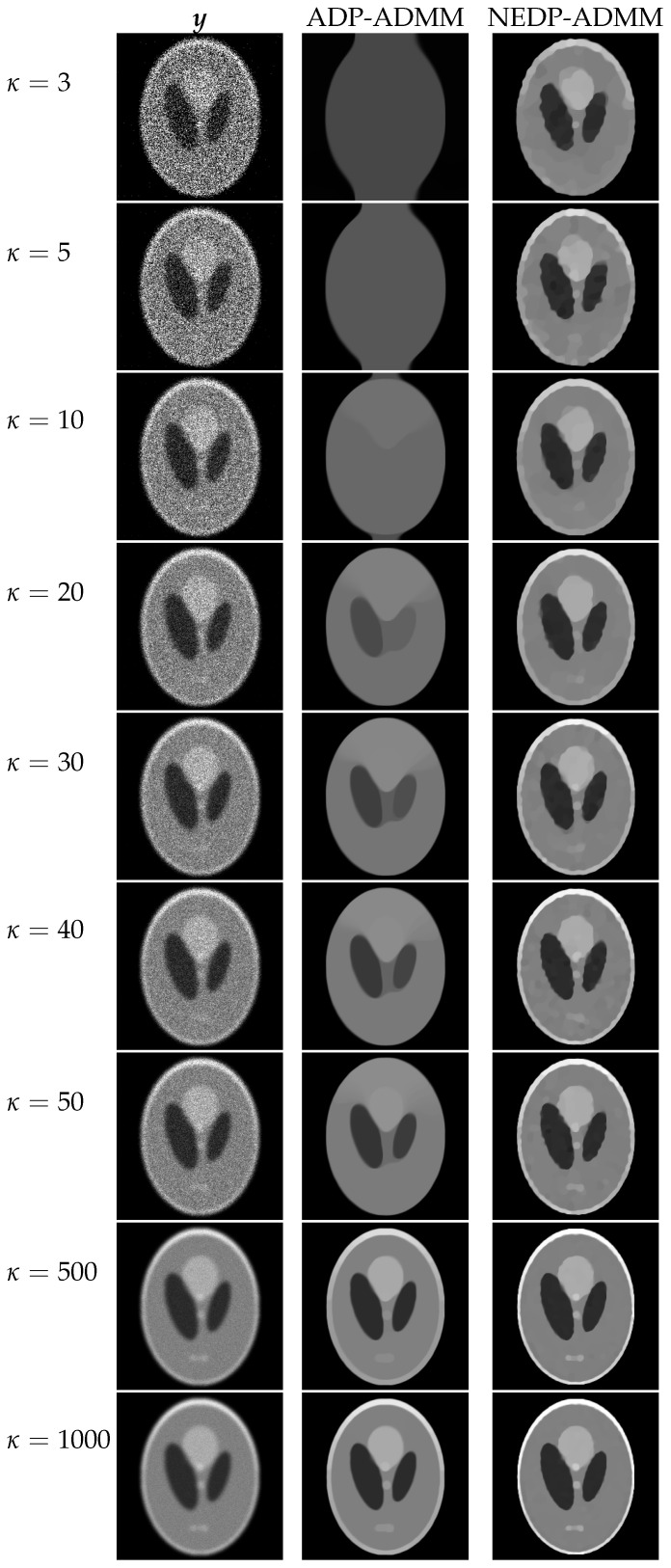
Test image phantom. **Left** column: observed data y corrupted by Gaussian blur with parameters band = 13, sigma = 3 and Poisson noise with different κ-values ranging from 3 to 1000. **Middle** column: restorations by ADP-ADMM. **Right** column: restorations by NEDP-ADMM.

From the restorations shown in Figure 13 and Figure 14, one can also notice that the slight improvement in terms of SSIM does not correspond to any significant visual improvements. In fact, along the whole range of considered photon counts κ, the NEDP-ADMM is capable of returning sharper restorations. This reflects the tendency of ADP-ADMM applied on the current test image in selecting not sufficiently large μ-values, so that, in the TV-KL, the regularization term takes over. We also remark that for the current test image, the SSIM value does not seem to be particularly meaningful.

**Figure 15:**
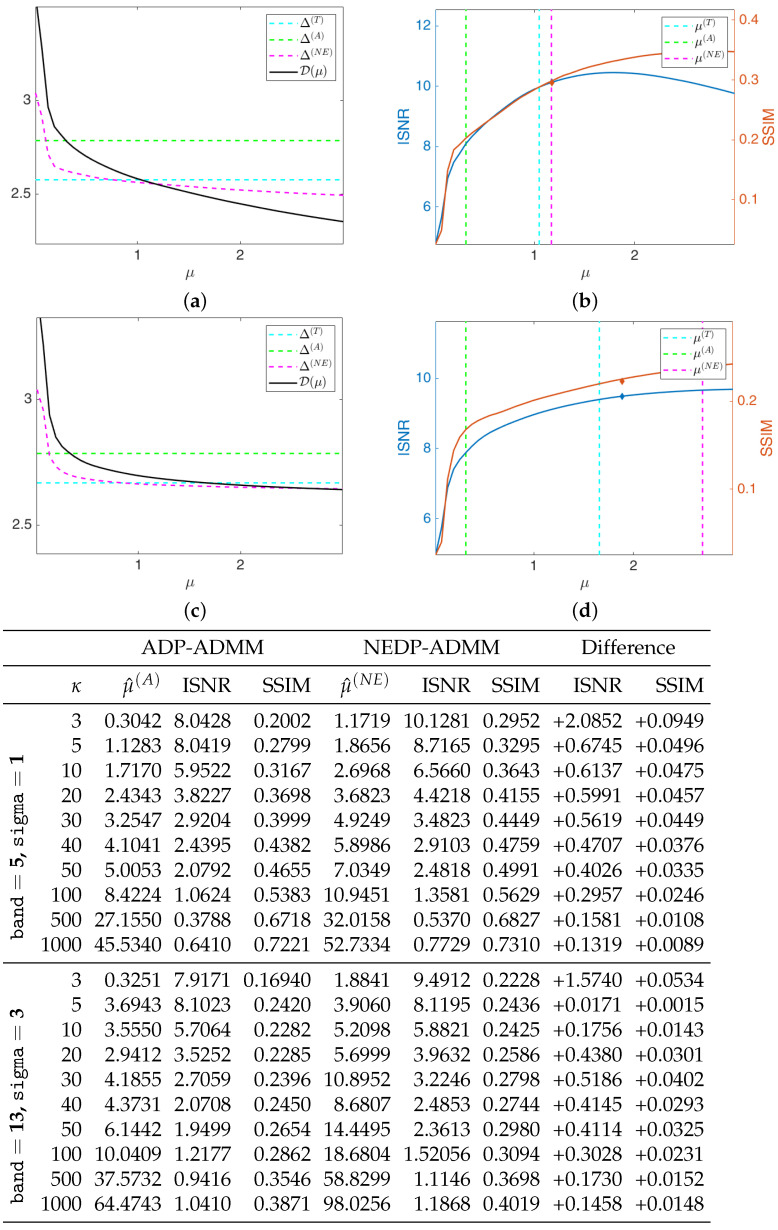
Test image cells. **Top**: discrepancy curve divided by $10^{4}$ (**a**,**c**) and ISNR/SSIM values (**b**,**d**) achieved for different μ-values with κ=3 and Gaussian blur with parameters band = 5, sigma = 1 (**first** row) and band = 13, sigma = 3 (**second** row). *Bottom*: output μ-values and ISNR/SSIM values obtained by the ADP-ADMM and the NEDP-ADMM for the two blur levels considered and different photon counts κ.

For the fourth and final test image, cells, we show in Figure 15 the behavior of the discrepancy function D(μ;y), as well as of the ISNR and SSIM values in the *a posteriori* framework for the two blur levels and κ=3. Note that, in the *a posteriori* setting, for both blur levels, the NEDP achieves higher ISNR and SSIM values when compared to the ADP. However, we observe that when considering the larger blur level, the output ${\hat{\mu }}^{\left(NE\right)}$ of the NEDP-ADMM is smaller than $\mu^{\left(NE\right)}$; this behavior can be ascribed, once again, to the ADMM convergence issues and the different values selected for the penalty parameters.

From the values reported in the bottom part of Figure 15, we notice that the NEDP-ADMM outperforms the ADP-ADMM in every photon count regime. Clearly, the closer ${\hat{\mu }}^{\left(A\right)}$ and ${\hat{\mu }}^{\left(NE\right)}$, the smaller the difference in terms of ISNR and SSIM.

**Figure 16:**
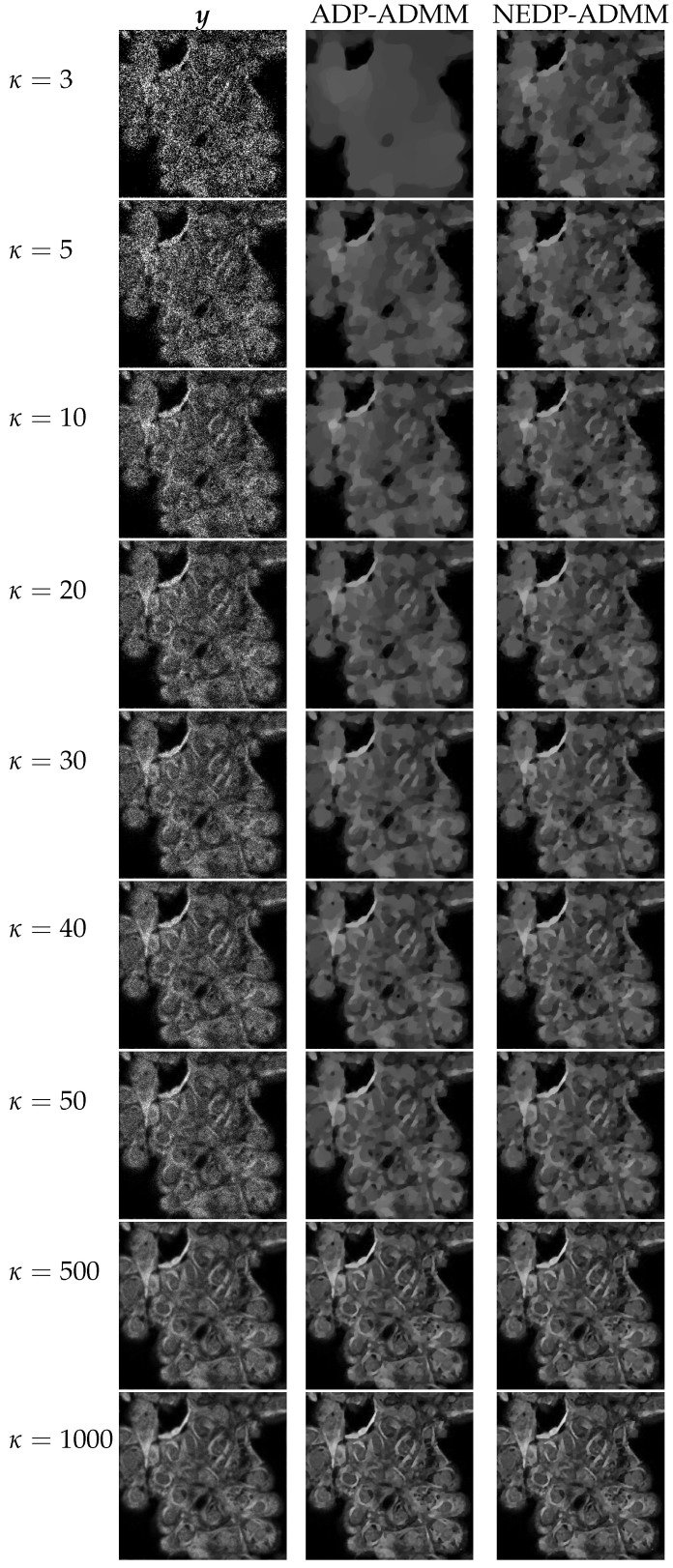
Test image cells. **Left** column: observed data y corrupted by Gaussian blur with parameters band = 5, sigma = 1 and Poisson noise with different κ-values ranging from 3 to 1000. **Middle** column: restorations by ADP-ADMM. **Right** column: restorations by NEDP-ADMM.

**Figure 17:**
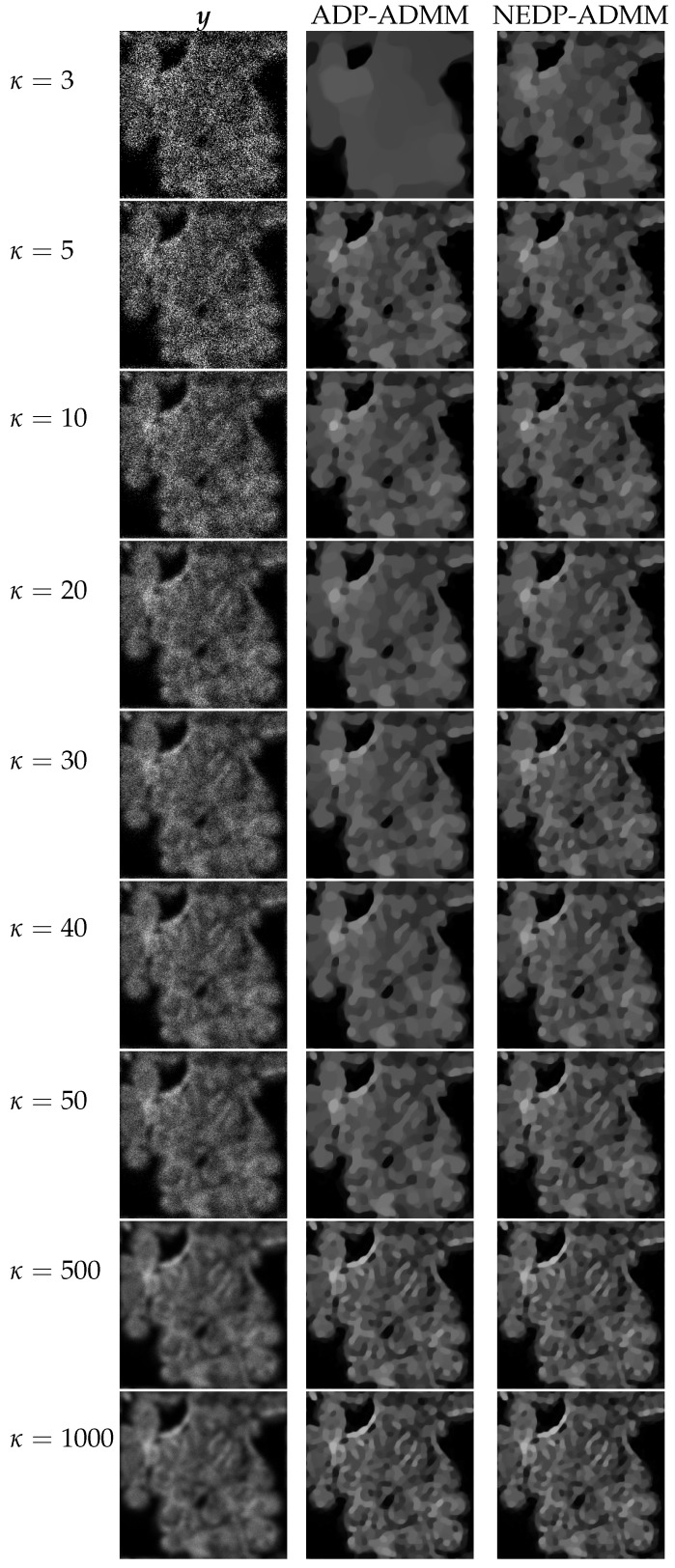
Test image cells. **Left** column: observed data y corrupted by Gaussian blur with parameters band = 13, sigma = 3 and Poisson noise with different κ-values ranging from 3 to 1000. **Middle** column: restorations by ADP-ADMM. **Right** column: restorations by NEDP-ADMM.

The restorations computed by the ADP-ADMM and the NEDP-ADMM are shown in Figure 16 for the smaller blur level and in Figure 17 for the larger one. The obtained results confirm the values reported in the bottom of Figure 15. Moreover, also from a visual viewpoint, the difference between the two performances increases when going from high- to low-count regimes.

## 6. Conclusions and Future Work

We propose an automatic selection strategy for the regularization parameter of variational image restoration models under Poisson noise corruption based on a nearly exact version of the approximate discrepancy principle originally proposed in [5]. Our approach relies on Monte Carlo simulations, which have been designed with the purpose of providing meaningful insights into the limitations of the original approximate strategy, especially in the low-count Poisson regime. The proposed version of the discrepancy principle has then been derived by means of a weighted least-square fitting and embedded along the iterations of an efficient ADMM-based optimization scheme. Our approach has been extensively tested on different images and for different photon count values, ranging from very low to high values. When compared to the original approximate selection criterion, the proposed strategy has been shown to drastically improve the quality of the output restorations in low-count regimes and in mid-count/high-count regimes on images characterized by few large pixel values.

From an analytical point of view, investigating the uniqueness of the regularization parameter value satisfying the proposed discrepancy principle will certainly constitute future work. From a modeling and applicative perspective, the effectiveness of the proposed approach when applied to variational models containing regularizers other than TV or aimed at solving inverse problems other than image restoration will be the subject of future analysis. Finally, from an algorithmic viewpoint, a matter that deserves further investigation is the (possibly automatic) selection of the three ADMM penalty parameters, which can significantly affect the speed of convergence of the numerical solution scheme. 

## Data Availability

Not applicable.
